# Sex and age specific bone mineral density trends in Sri Lankan adults support the need for normative reference data

**DOI:** 10.3389/fendo.2026.1748490

**Published:** 2026-03-04

**Authors:** Muhundhapriya Varatharajan, Sachith P. Abeysundara, Mohan Lal Jayatilake, Sachith Abhayaratna, Padma Badra Hewavithana

**Affiliations:** 1 Department of Radiology, Faculty of Medicine, University of Peradeniya, Peradeniya, Sri Lanka; 2 Department of Statistics and Computer Science, Faculty of Science, University of Peradeniya, Peradeniya, Sri Lanka; 3 Department of Radiography and Radiotherapy, Faculty of Allied Health Sciences, University of Peradeniya, Peradeniya, Sri Lanka; 4 Durdans Hospital, Colombo, Sri Lanka

**Keywords:** BMD, dual energy X-ray absorptiometry, healthy population, normative, Sri Lanka

## Abstract

**Introduction:**

Bone Mineral Density (BMD) is a critical determinant of osteoporosis diagnosis and fracture risk. Absence of normative BMD data for Sri Lankans has necessitated reliance on Caucasian references, potentially misestimating the BMD. This study aimed to establish age-related BMD trends for optimal bone health management.

**Methods:**

A retrospective correlational analysis was conducted on 10,946 adults (4,005 men, 6,941 women; 21–80 years) who underwent lumbar spine and hip scans using Hologic Dual Energy X-ray absorptiometry at five private health institutions in Western Province. Individuals with medical conditions or treatments known to affect bone metabolism were excluded.

**Results:**

BMD values showed strong positive correlations across lumbar spine and hips (r = 0.61 - 0.94, p < 0.001) with negligible bias between hips (mean difference ≈ 0.003 g/cm2). Minor right - left hip differences appeared only in 21– 30 age group (p < 0.05). Males had consistently higher BMD than females. In females, lumbar BMD peaked at 31–40 years (0.980 g/cm2) and declined thereafter (F = 279.76, p < 0.001; η² = 0.156); hip BMD peaked at 41–50 years (~0.95 g/cm2) and declined after 50 years (~0.04–0.06 g/cm2 /decade). In males, lumbar BMD peaked at 31–40 years (0.997 g/cm2) with modest decline (F = 4.73, p < 0.001; η² = 0.006), while hip BMD remained stable until 60, then decreased (~0.03 g/cm2 /decade). Contralateral hip BMD showed strong symmetry, supporting the reliability of single-hip measurements in clinical practice. Higher body mass index was positively associated with BMD in both sexes, particularly in women. Years since menopause accounted for a substantial portion of variance (15%-19%) in BMD, with trabecular-rich lumbar spine exhibiting faster early loss compared to cortical-rich hips, which declined more gradually in later decades. Compared with Caucasian reference data, Sri Lankan participants displayed consistently lower BMD, with the greatest deficits observed in postmenopausal women (up to 15% lower), highlighting the need for population-specific reference ranges.

**Discussion:**

These findings demonstrate the need for population-specific BMD reference values to improve diagnostic accuracy and guide clinical management of osteoporosis and osteopenia, particularly in postmenopausal women and older adults.

## 1 Introduction

Bone mineral density (BMD) is a measurement of the amount of minerals, primarily calcium and phosphorus, present in an individual’s bones, which is a critical indicator of bone health (1). In Dual Energy X-ray Absorptiometry (DXA), BMD is reported as areal BMD, calculated by dividing the aggregate bone mineral content (BMC) by the scanned area, and expressed in grams per square centimeter (g/cm^2^). BMD in different populations is determined by numerous factors such as age, gender, ethnicity, race, genetics, nutritional level, lifestyles, medical conditions etc. (2–5).

BMD measurement plays a crucial role in assessing the risk of getting a fracture (6), monitoring the efficacy of the treatments for osteoporosis (7–10), orthopedic surgical planning (11, 12), and evaluating overall bone health (13). Low BMD is associated with an increased likelihood of fragility fractures, particularly in post-menopausal females and older males (14). While osteoporosis and fragility fractures are often emphasized in females, males also face significant risks, with studies indicating that approximately 20–25% of hip fractures globally occur in men, and men have a higher mortality rate following such fractures, compared to women due to greater frailty and comorbidities (15).

The spectrum of BMDs as per the age and gender of a large random sample of a certain community is defined as the normative BMD data of the specific community (16). This data is derived from extensive research, large-scale studies and it summarizes the BMD values considered typical or average for that specific population (16, 17). DXA analysis methods, use reference values from the United States of America or Northern European population by default, causing potential discrepancies and biases when used to interpret BMD of different populations with distinct genetic, geographic, and socioeconomic traits. Currently, DXA scanners in Sri Lanka rely on reference values derived from the National Health and Nutritional Examination Survey (NHANES) database, which predominantly represents Caucasian populations. This reliance introduces significant diagnostic bias, due to ethnic and geographic differences in bone structure and mineralization. Hence Sri Lankans may be misclassified, either underdiagnosed or over diagnosed. Hence to ensure precise diagnosis, it’s imperative to compare an individual’s BMD against the T-score defined for the specific population to which that particular individual belongs.

Population-specific biological and environmental factors may further influence BMD and limit the applicability of non-local reference standards. In this context, approximately 90% of Sri Lankan adults have been reported to have inadequate levels of vitamin D (18, 19), yet vitamin D insufficiency remains a relatively less addressed public health issue in Sri Lanka. As vitamin D plays a critical role in calcium absorption and metabolism, its insufficiency can directly affect BMD, further emphasizing the need for population-specific normative BMD reference values.

Numerous studies have been conducted worldwide to establish population-specific normative BMD data. Most of the studies were aimed at demonstrating the considerable differences in the prevalence of osteoporosis, when population-specific BMD reference data is used instead of the reference values recognized for white populations (13, 20–25). Most of the Sri Lankan community-based studies have been carried out to determine the prevalence of osteoporosis by estimating age-specific BMD. Despite the efforts made by previous research, the Sri Lankan scientific community is still in need of the properly established central BMD normative data. Currently in Sri Lanka, clinicians have to compare the BMD of Sri Lankans against Caucasian reference values for BMD because of the unavailability of normative central BMD data for the Sri Lankan population (26–28). Therefore, there is a great need for normative BMD data for both males and females in Sri Lanka, to provide a scientific basis for optimal bone health management.

This study aimed to establish BMD reference values for the lumbar spine and total hip across different age groups in Sri Lankan adults aged 21 to 80 years. By examining age-, sex-, body mass index (BMI), and menopause-related trends in BMD, the study sought to fill a critical gap in population-specific skeletal health data. Specifically, the objectives were to determine BMD across different age categories for both males and females separately, thereby enabling age specific comparisons and evaluations and to compare BMD values from the current study with existing reference standards.

## 2 Materials and methods

### 2.1 Study setting and study design

This was a retrospective multicenter study using DXA scan data collected from January 2015 to December 2024 from five private healthcare institutions in the Western province. The study population consisted of male and female patients who visited the hospitals for screening as a part of preventive health check-ups between the age of 21–80 years. Data was utilized for all patients who underwent lumbar spine and hip scans during the same scan session.

The Ethics Review Committee of the Faculty of Medicine, University of Peradeniya, which is approved by the Forum of Ethics Review Committees of Sri Lanka (FERCSL), determined that this study was exempt from ethical review as it used anonymized retrospective data. Additionally, the participating hospitals’ Institutional Review Boards (IRBs) granted institutional authorization. The rights of human beings, confidentiality, and privacy were all fully respected.

### 2.2 Exclusion criteria

Participant exclusions were determined based on responses to a standardized self-administered questionnaire, which captured demographic details, health status, medication use, and reproductive history. Scans from individuals diagnosed with, or receiving treatment for, osteoporosis or osteopenia, as well as those with pathological, medical, or surgical conditions known to affect normal bone metabolism, were excluded. Osteopenia was defined based on both responses to a standardized self-administered questionnaire (prior clinical diagnosis) and also from DXA-derived T-scores (−1.0 to −2.5). This exclusion was applied as a precautionary measure to establish normative BMD reference values reflecting optimal bone health. Although the exclusion of individuals with osteopenia may limit the full representativeness of the general population, the primary objective of this study was to derive age- and sex-specific normative BMD reference values based on healthy bone status. Inclusion of osteopenia individuals, who may already exhibit altered bone metabolism or be receiving pharmacological interventions to prevent progression to osteoporosis, could introduce systematic bias and lead to underestimation of true normative BMD values. Therefore, their exclusion was intended to ensure methodological validity and accuracy of the normative dataset. In addition, participants using below listed medications or presenting with medical conditions that could alter bone metabolism were also excluded from the study.

- On medications that may alter normal bone metabolism. Such as Glucocorticoids, Risedronate, Ibandronate, Raloxifene, Parathyroid hormone, Alendronate, Hormone replacement therapy (HRT), Calcitonin, Strontium ranelate, Zoledronate, Denosumab etc.
- Diagnosed with pathologies or medical conditions that may alter normal bone metabolism. Such as Anorexia, Bulimia, Asthma, Any seizure disorders, Cancer, Inflammatory bowel disease, Renal disease, Hyperparathyroidism etc.
- Diagnosed with diseases that may alter the gross morphology of the scan sites. For example, Wedge fractures in scan region, Femoral neck fractures, Excessive osteophyte formation, Severe scoliosis.
- Had prostheses or artifacts are in place, that may cause the BMD measurement to be inaccurate, such as Hip replacement and Bone cement in vertebral bodies.

**Figure 1:**
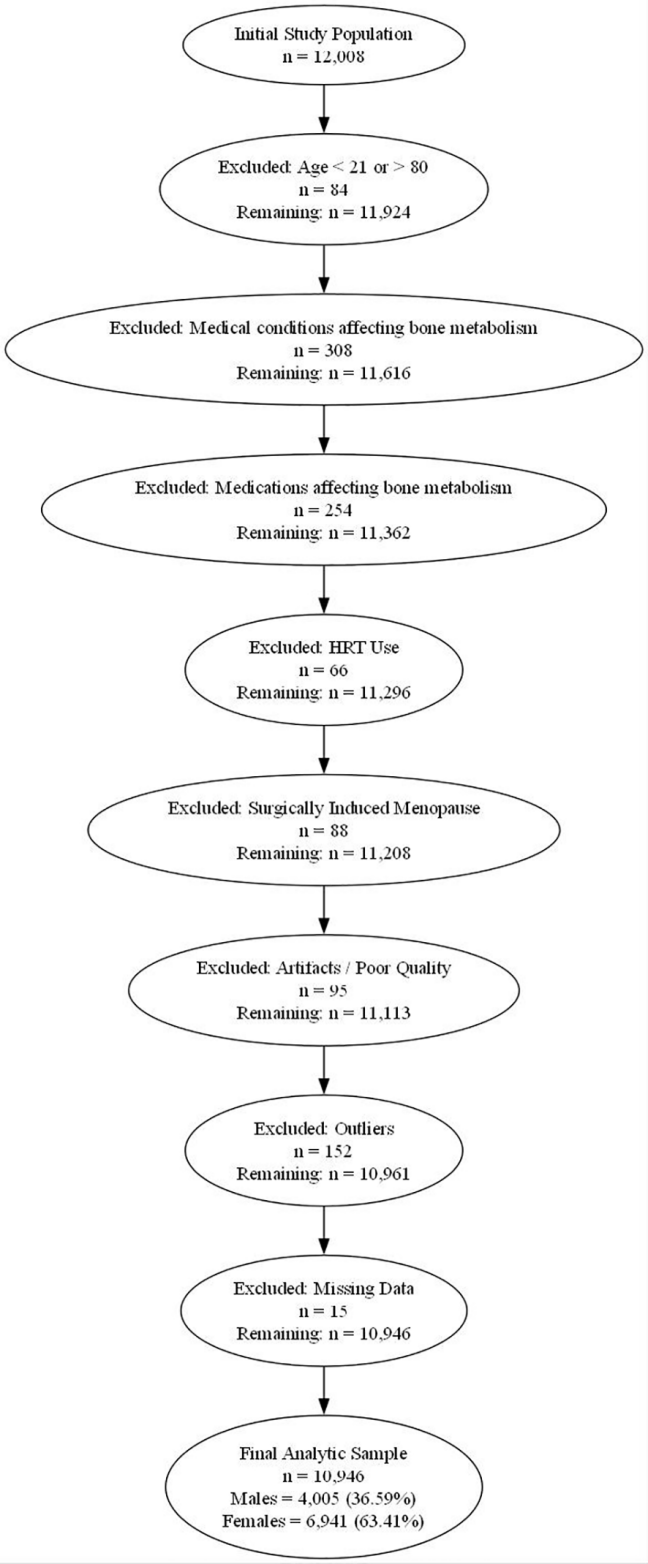
STROBE flow chart of the study population. Flowchart depicting the sequential exclusion criteria applied to derive the final sample from an initial pool of 12,008 collected scans. Participants were excluded based on age criteria (n = 84), medical conditions affecting bone metabolism (n = 308), medications affecting bone metabolism (n = 254), HRT (n = 66), surgically induced menopause (n = 88), artifacts or poor scan quality (n = 95), statistical outliers (n = 152), and missing data (n = 15). After applying all exclusion criteria, the final analytic sample consisted of 10,946 individuals, including 4,005 males (36.59%) and 6,941 females (63.41%).

Furthermore, women who were receiving HRT at the time of their DXA scan, or who had discontinued HRT within the preceding 12 months, were excluded to minimize its confounding effect on BMD. Additionally, women with surgically induced menopause, defined as cessation of menstruation due to bilateral oophorectomy, with or without hysterectomy were excluded to maintain consistency with natural menopausal profiles in the reference population. Vertebrae demonstrating a T-score difference greater than 1.0 compared with adjacent vertebral levels were also excluded from the analysis in accordance with International Society for Clinical Densitometry (ISCD) recommendation. A flowchart summarizing the process of sample selection is presented in **Figure 1**.

### 2.3 Study instrument and DXA scan procedure

The study used Hologic DXA scanners across five sites: Site 1 – Horizon Wi (APEX 5.6.0.5); Site 2 – Discovery W (APEX 3.3.0.1); Site 3 – Discovery W (APEX 3.2); Site 4 – Discovery W (APEX 3.3.0.1); and Site 5 – Horizon W (APEX 5.6.0.4). Scan data were retrieved from institutional DXA systems with no patient identifiers. Only the entries of subjects with available duly filled questionnaires were used in this study. The questionnaire used is a standard tool used in the institutions that have been provided by Hologic manufacturers (**Supplementary Material**).

All scans followed manufacturer protocols for acquisition and analysis. Lumbar spine BMD was evaluated according to the ISCD Official Positions (29), using L1–L4 (anteroposterior view) as the region of interest. The total proximal femur served as the hip region of interest. As all sites routinely performed bilateral hip scans, both sides were analyzed.

### 2.4 Methods and DXA standardization

To ensure inter-site comparability, cross-calibration was performed using an anthropomorphic spine phantom, scanned five times on each device under identical acquisition conditions. The mean total BMD values obtained from each site were compared, and the inter-device offsets were found to be within ±1%, which is consistent with the ISCD recommendations for acceptable cross-calibration accuracy. Therefore, no correction factors were applied to the BMD results. All scanners operated in the standard scan mode using a fan-beam X-ray configuration. Furthermore, it was ensured that during the study period phantom measurements for quality control checks showed stable results. *In-vivo* precision testing (repeat, fully repositioned scans on the same day) was not feasible for this study because the data were collected retrospectively, which precluded the recall of participants for additional scans, and no routine protocol of paired repeat patient scans was performed during the data collection period. All participants had their standing height and weight measured. The BMI was calculated by dividing weight in kilograms by height in meters squared.

### 2.5 Reference database

For international comparison, normative BMD values were benchmarked against the NHANES 2005–2008 reference Table 1 and 16 (30). In our dataset, the number of participants aged exactly 20 years was very small, which limited statistical reliability. To ensure adequate sample size per category, we therefore redefined the age bands as 21-30, 31-40, 41-50, 51-60, 61-70, and 71–80 years. This recategorization provided more balanced group sizes and improved the statistical stability of comparisons with the reference database.

### 2.6 Statistical analysis

Subjects were grouped into 10-year age categories for cross-sectional analysis. Continuous variables are presented as mean ± standard deviation (SD). Correlations between BMD (lumbar spine, right hip, and left hip) and physiological factors (age, weight, height, BMI, and years since menopause in women) were assessed using Spearman’s rank correlation analysis. Differences in BMD between right and left hips were evaluated using paired-sample t-tests.

Differences in BMD across age groups, BMI categories, and years since menopause were assessed using one-way analysis of variance (ANOVA). Assumptions of normality and homogeneity of variance were evaluated, and Welch’s ANOVA was applied when these assumptions were violated. *Post hoc* pairwise comparisons were performed using the Games–Howell test. Effect sizes were quantified using eta squared (η^2^) for ANOVA-based analyses and Hedges’ g for pairwise comparisons.

Agreement between right and left hip BMD measurements was assessed using Bland–Altman analysis. Comparisons between cohort BMD values and NHANES reference data were performed using independent-sample t-tests stratified by age and sex. Smoothed age- and sex-specific BMD centile curves were generated using Generalized Additive Models (GAMs). Bootstrap resampling was used to validate selected estimates. Statistical significance was set at p < 0.05. Data was processed and analyzed in Python (version 3.12.4) using standard statistical libraries.

## 3 Results

### 3.1 Demographic analysis

**Table 1** summarizes the demographic and anthropometric characteristics of the study population (n = 10,946; 4,005 males [36.59%], 6,941 females [63.41%]). The largest proportion in both sexes was in the 51–60 age group, followed by 61–70 years. The mean age indicated minimal variations between sexes: males 57.87 ± 2.8 years, females 60.04 ± 2.9 years. In both sexes, the highest mean weight occurred at 41–50 years, with a subsequent gradual decline. Maximum mean height was observed at 31–40 years. BMI peaked in middle age, 41–60 years, and then decreased in older age groups, with females showing slightly higher values compared to males. Among women, 1,254 participants (18.1%) were estimated as premenopausal, while 5,687 (81.9%) were estimated as postmenopausal, indicating a predominantly postmenopausal cohort.

**Table 1:** Demographic and anthropometric characteristics of study participants by age group and sex, including mean age, weight, height, and body mass index (BMI) ± standard deviation (SD).

Age category	Number of subjects		Mean age (years) (± SD)		Mean weight (kg) (± SD)		Mean height (cm) (± SD)		Mean BMI kg/m^2^ (± SD)	
	Male	Female	Male	Female	Male	Female	Male	Female	Male	Female
21-30	58	60	27.39 (2.74)	26.96 (2.73)	70.74 (12.59)	59.97 (12.00)	168.97 (6.95)	156.53 (7.86)	24.76 (4.08)	24.43 (4.28)
31-40	215	254	36.02 (2.92)	36.16 (2.93)	73.44 (11.91)	62.92 (11.18)	169.16 (6.80)	157.25 (6.60)	25.61 (3.62)	25.45 (4.28)
41-50	414	702	46.39 (3.13)	46.62 (2.92)	74.55 (10.34)	65.00 (10.51)	167.88 (6.55)	155.86 (6.49)	26.44 (3.38)	26.74 (3.95)
51-60	1786	2485	55.57 (2.80)	55.80 (2.88)	70.98 (10.85)	63.45 (10.14)	164.78 (6.37)	153.31 (6.15)	26.12 (3.60)	26.98 (3.94)
61-70	1060	2348	65.00 (2.77)	65.26 (2.84)	69.20 (10.92)	60.09 (10.12)	163.93 (6.24)	151.23 (6.25)	25.73 (3.67)	26.25 (4.03)
71-80	472	1092	74.32 (2.61)	74.42 (2.63)	65.54 (10.70)	57.20 (9.79)	162.11 (6.15)	149.44 (6.26)	24.91 (3.65)	25.60 (4.06)

### 3.2 Descriptive statistics

In male subjects, all the BMD sites revealed considerable positive correlations. Lumbar spine BMD was strongly correlated with the BMD values of right hip (ρ = 0.613, p < 0.001), as well as the left hip (ρ = 0.615, p < 0.001). Similarly, values for right and left hip BMD were strongly correlated with each other (ρ = 0.936, p < 0.001), supporting parallel bone density patterns in axial sites. In female subjects, the relationships were likewise strong, such that lumbar spine BMD exhibited significant relationships with that of the right hip BMD (ρ = 0.684, p < 0.001), as well as with that of the left hip BMD (ρ = 0.693, p < 0.001). The correlation between BMD of the right hip with left hip was particularly strong (ρ = 0.938, p < 0.001), suggesting high anatomical concordance in hip BMD values.

The high correlation coefficients suggest a high consistency of measurement between both hips. Bland–Altman analysis confirmed negligible systematic bias, with mean differences near zero (males = 0.0034 g/cm^2^, limits −0.0798 to 0.0865; females = 0.0029 g/cm^2^, limits −0.0871 to 0.0930) (**Supplementary Table 1**; **Supplementary Figures 1**, **2**). Paired t-tests were also utilized in order to examine differences in BMD between the hip of the right side and the hip of the left side in all age groups, with males and females being examined individually. Statistically significant differences were identified only in the 21–30 age group for both sexes (p < 0.05), with no significant differences in older groups (p > 0.05).

#### 3.2.1 Lumbar spine BMD

Across all age groups, lumbar spine BMD was higher in males than females. Among females, BMD correlated positively with weight (ρ = 0.419, p < 0.001), height (ρ = 0.317, p < 0.001), and BMI (ρ = 0.291, p < 0.001), and negatively with age (ρ = −0.400, p < 0.001) and years since menopause (ρ = −0.412, p < 0.001), indicating progressive postmenopausal bone loss. Mean lumbar BMD peaked at ages 31–40 years (0.980 g/cm^2^) and reached its lowest at 71–80 years (0.793 g/cm^2^), with the greatest loss between the fifth and sixth decades (0.0996 g/cm^2^ per decade). After which the decline persisted but was less pronounced in the subsequent decades. There were statistically significant ANOVA differences in lumbar BMD with age groups (F = 279.76, p < 0.001), with a moderate effect size (η^2^ = 0.156). Games–Howell tests confirmed significant pairwise differences, particularly between 31–40 and 71–80 years (mean = 0.187 g/cm^2^; Hedges’ g = 1.27), but none among 21 to 50 years (p > 0.85; g < 0.1).

In males, lumbar BMD was weakly positively associated with weight (ρ = 0.272, p < 0.001), height (ρ = 0.159, p < 0.001), and BMI (ρ = 0.217, p < 0.001) and showed a weak negative association with age (ρ = −0.042, p < 0.01). The peak BMD was seen in the age group of 31–40 years (0.997 g/cm^2^) and gradually declined reaching the lowest in 71–80 years (0.959 g/cm^2^). A more noticeable reduction occurred between fifth and sixth decades (0.022 g/cm^2^ per decade) with a trivial increase after 60 years (+0.006 g/cm^2^ per decade). While male lumbar BMD declines with age, the pattern is less steep, and more variable compared to females. Consequently, Welch’s ANOVA showed statistically significant difference in lumbar BMD across age groups in men (F = 4.73, p < 0.001). However, the effect size analysis indicated that age group accounted for only a small proportion of variance in men (η^2^ = 0.0057), with small *post-hoc* (Games-Howell test) differences between most age groups (Hedges’ g ≤ 0.26), suggesting age-related changes in lumbar BMD were modest in men. In women, age had a moderate effect size (η^2^ = 0.156) with large *post-hoc* differences (Hedges’ g ≤ 1.28), indicating more pronounced age-related changes in lumbar BMD.

#### 3.2.2 Hip BMD

Both right and left hip BMD showed comparable correlation patterns across sexes. In males, right hip BMD correlated positively with weight (ρ = 0.359, p < 0.001) and BMI (ρ = 0.354, p < 0.001); left hip BMD showed similar relationships with weight (ρ = 0.375, p < 0.001) and BMI (ρ = 0.362, p < 0.001). Age correlated weakly and negatively (ρ = −0.087 to −0.103, p < 0.01), while height showed only minor positive associations (ρ = 0.091–0.105, p < 0.001). In females, hip BMD was strongly associated with weight and BMI (right: ρ = 0.493, 0.410; left: ρ = 0.501, 0.415; all p < 0.001) and weakly with height (ρ ≈ 0.25, p < 0.001). Age and years since menopause correlated negatively (ρ = −0.406 to −0.420, p < 0.001).

Males showed consistently higher hip BMD than females. In women, BMD showed a modest rise in early adulthood, reaching a maximum mean BMD in the age range 41–50 years (right = 0.948; left = 0.945 g/cm^2^) and declined after 50 years, with losses of 0.043–0.046 g/cm^2^ per decade. This BMD loss further accelerated to 0.0692 g/cm^2^ and 0.0660 g/cm^2^ per decade for the right and left hips, respectively, reaching the lowest values in the 71–80 age group (right hip: 0.786 g/cm^2^; left hip: 0.784 g/cm^2^). In males, a stable pattern of hip BMD was observed across age groups, with small declines noted in later decades. The highest mean BMD was seen in the 51–60 age category (right hip: 0.984 g/cm^2^; left hip: 0.981 g/cm^2^), while the lowest was in the 71–80 age group (right hip: 0.942 g/cm^2^; left hip: 0.935 g/cm^2^). Both right and left hips showed similar trends, with the greatest BMD loss per decade being around 0.029 g/cm^2^ after the sixth decade, which was less than the decline seen in females.

**Figure 2:**
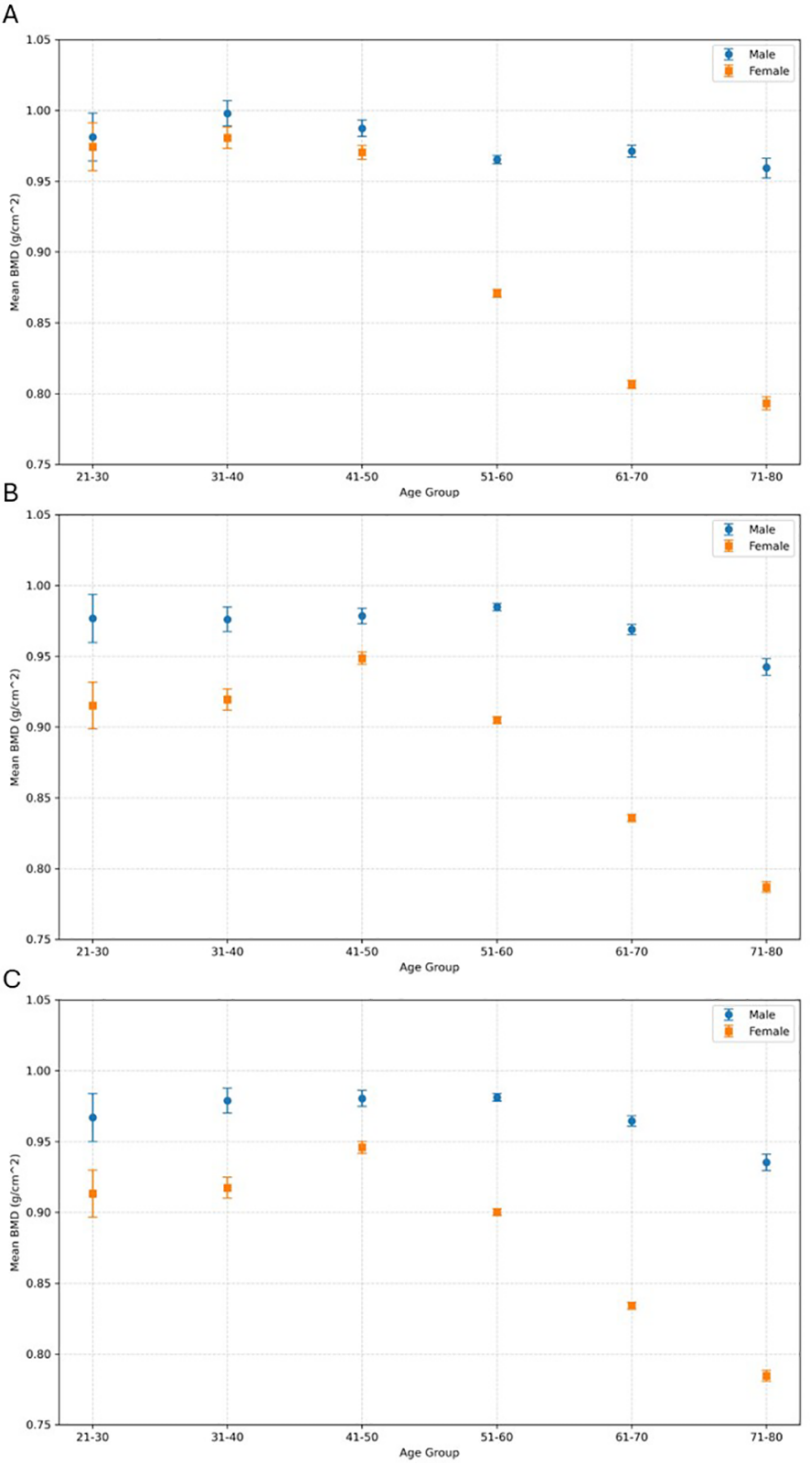
**(A)** Lumbar Spine, **(B)** Right Hip, **(C)** Left Hip mean bone mineral density (BMD) trend across age groups, stratified by sex. Error bars represent the 95% confidence intervals.

Normality held for males across all age groups but not for females, above 51 years. Welch’s ANOVA revealed significant age-related differences in both sexes (females-right hip: F = 257.14, p < 0.001, η^2^ = 0.154; left hip: F = 243.44, p < 0.001, η^2^ = 0.149; males - right hip: F = 9.13, p < 0.001, η^2^ = 0.0127; left hip: F = 11.61, p < 0.001, η^2^ = 0.0156), with larger declines in women. **Figures 2A–C** presents the interval plots with 95% confidence intervals visually supporting these trends, showing BMD reduction with age. **Table 2** shows the percentile distribution of BMD across age group and by gender. Additionally, the reference curves derived using GAMs, along with the age and sex specific smoothed centile reference values (P5–P95) for lumbar spine and bilateral hip BMD from 21 to 80 years in one-year intervals, are provided in the Supplementary Materials (**Supplementary Figures 3A–F**).

**Table 2:** Bone mineral density means, standard deviations, and percentile values (5th–95th) for males and females across age categories.

Region	Gender	Age group	Sample size	Mean BMD	Std Dev	Percentile								
						5^th^	10^th^	15^th^	25^th^	50^th^	75^th^	85^th^	90^th^	95^th^
Lumbar spine	Female	21–30	60	0.974	0.130	0.752	0.808	0.863	0.905	0.973	1.066	1.108	1.122	1.182
		31–40	254	0.980	0.119	0.772	0.826	0.854	0.905	0.979	1.067	1.096	1.132	1.174
		41–50	702	0.970	0.128	0.776	0.810	0.837	0.887	0.964	1.057	1.110	1.140	1.187
		51–60	2485	0.870	0.137	0.660	0.700	0.731	0.776	0.861	0.961	1.017	1.055	1.109
		61–70	2348	0.806	0.137	0.592	0.636	0.663	0.709	0.800	0.892	0.948	0.986	1.047
		71–80	1092	0.793	0.152	0.567	0.614	0.638	0.688	0.780	0.879	0.949	0.987	1.068
	Male	21–30	58	0.981	0.128	0.727	0.817	0.855	0.905	0.979	1.071	1.108	1.142	1.199
		31–40	215	0.997	0.130	0.798	0.832	0.862	0.902	0.997	1.085	1.118	1.160	1.240
		41–50	414	0.987	0.120	0.795	0.831	0.861	0.908	0.986	1.063	1.112	1.132	1.187
		51–60	1786	0.965	0.124	0.765	0.806	0.834	0.879	0.960	1.051	1.097	1.130	1.174
		61–70	1060	0.971	0.137	0.748	0.795	0.833	0.878	0.965	1.061	1.127	1.157	1.205
		71–80	472	0.959	0.152	0.712	0.769	0.802	0.853	0.946	1.060	1.120	1.174	1.228
Right Hip	Female	21–30	60	0.915	0.128	0.659	0.721	0.802	0.847	0.935	0.987	1.045	1.050	1.092
		31–40	254	0.919	0.118	0.724	0.778	0.801	0.851	0.917	1.004	1.043	1.072	1.111
		41–50	702	0.948	0.113	0.756	0.800	0.829	0.875	0.951	1.026	1.066	1.090	1.127
		51–60	2485	0.904	0.119	0.715	0.757	0.782	0.823	0.902	0.983	1.028	1.063	1.106
		61–70	2348	0.835	0.122	0.640	0.680	0.708	0.751	0.832	0.916	0.958	0.992	1.045
		71–80	1092	0.786	0.121	0.603	0.634	0.659	0.698	0.777	0.864	0.908	0.944	0.997
	Male	21–30	58	0.976	0.129	0.772	0.806	0.835	0.883	0.973	1.082	1.119	1.136	1.150
		31–40	215	0.976	0.128	0.754	0.792	0.830	0.882	0.989	1.059	1.110	1.138	1.178
		41–50	414	0.978	0.110	0.802	0.844	0.869	0.901	0.971	1.060	1.093	1.120	1.156
		51–60	1786	0.984	0.112	0.798	0.840	0.863	0.906	0.984	1.064	1.100	1.130	1.167
		61–70	1060	0.968	0.119	0.784	0.824	0.843	0.885	0.971	1.045	1.093	1.130	1.171
		71–80	472	0.942	0.130	0.744	0.774	0.800	0.846	0.937	1.043	1.081	1.108	1.150
Left Hip	Female	21-30	60	0.913	0.128	0.690	0.720	0.790	0.828	0.930	0.994	1.028	1.042	1.096
		31-40	254	0.917	0.116	0.715	0.780	0.809	0.838	0.916	0.986	1.044	1.063	1.098
		41-50	702	0.945	0.113	0.754	0.797	0.835	0.873	0.948	1.024	1.065	1.098	1.130
		51-60	2485	0.900	0.120	0.711	0.752	0.775	0.815	0.895	0.980	1.026	1.060	1.110
		61-70	2348	0.834	0.121	0.645	0.683	0.710	0.749	0.829	0.910	0.958	0.994	1.049
		71-80	1092	0.784	0.125	0.595	0.631	0.653	0.688	0.778	0.866	0.916	0.954	1.002
	Male	21-30	58	0.966	0.129	0.764	0.797	0.830	0.882	0.964	1.047	1.107	1.130	1.181
		31-40	215	0.978	0.129	0.748	0.791	0.825	0.894	0.988	1.065	1.109	1.144	1.188
		41-50	414	0.980	0.114	0.785	0.836	0.863	0.905	0.983	1.063	1.096	1.119	1.163
		51-60	1786	0.981	0.113	0.796	0.840	0.866	0.904	0.983	1.058	1.098	1.129	1.170
		61-70	1060	0.964	0.120	0.766	0.815	0.838	0.883	0.960	1.044	1.096	1.127	1.170
		71-80	472	0.935	0.127	0.739	0.769	0.801	0.843	0.930	1.033	1.077	1.104	1.141

#### 3.2.3 BMI stratified analysis

Stratification by BMI showed a consistent positive association between body mass and BMD in both sexes. Underweight individuals had the lowest mean lumbar BMD (males: 0.844 g/cm^2^; females: 0.715 g/cm^2^), whereas obese participants had the highest (males: 1.001 g/cm^2^; females: 0.917 g/cm^2^). In females, assumption checks indicated non-normality and unequal variances, mainly in older groups. Additionally, Welch’s ANOVA revealed significant differences across BMI categories (F = 200.3, p < 0.001, η^2^ = 0.079), and *post-hoc* analysis showed the greatest declines in normal-weight women, while overweight and obese women maintained higher, more stable BMD values. All BMI groups showed sharp reductions after age 50, most notably in underweight and normal-weight women.

In males, lumbar BMD also differed significantly by BMI categories (F = 65.43, p < 0.001, η^2^ = 0.047). Obese men had higher BMD than normal-weight (mean difference = 0.056 g/cm^2^; g = 0.43) and underweight (0.156 g/cm^2^; g = 1.22) participants, which is also confirmed by bootstrap validation. Normal-weight men showed a modest rise in BMD until 40 years, followed by a steady decline, whereas overweight and obese men remained stable, with slight increases after 60 years (≈0.015–0.027 g/cm^2^ per decade).

Analysis of hip BMD revealed parallel to those observed in the lumbar spine, with body mass strongly influencing BMD across age strata. In both sexes, underweight participants consistently had the lowest values, whereas overweight and obese groups maintained the highest. In women, across all BMI groups hip BMD increased in early adulthood and declined steadily after 50 years. In men, overweight and obese groups showed biphasic patterns with minor midlife gains after the fifth decade which followed by a decline. The increase was most notable in the obese groups. In contrast, the normal weight group showed a steady decline in hip BMD across age groups, with no apparent midlife peak. Statistical tests also confirmed these descriptive patterns. In women, Welch’s ANOVA indicated significant BMI-related differences in hip BMD (right hip: F = 432.74, p < 0.001, η^2^ = 0.154; left hip: F = 450.60, p < 0.001, η^2^ = 0.159). In men, the differences were also highly significant (right hip: F = 168.17, p < 0.001, η^2^ = 0.114; left hip: F = 177.37, p < 0.001, η^2^ = 0.118).

#### 3.2.4 BMD trends across years since menopause

Female participants were grouped by years since menopause into eight 5-year intervals: non-menopausal, 1–5, 6–10, 11–15, 16–20, 21–25, 26–30, and >30 years. Lumbar BMD declined steadily with menopausal duration, from 0.968 g/cm^2^ in non-menopausal women to 0.775 g/cm^2^ in more than 30 years since menopause, averaging a loss of 0.0041 g/cm^2^ per year. The greatest decline occurred within the first 5 years post-menopause (−0.0166 g/cm^2^/year), slowing progressively to −0.0094 g/cm^2^ at 6–10 years and −0.0042 g/cm^2^ by 16–20 years, stabilizing thereafter. Welch’s ANOVA indicated significant group differences (F = 223.16, p < 0.001; η^2^ = 0.186), and Games–Howell tests confirmed large pairwise effects (e.g., Hedges’ g = 1.44 between non menopausal and >30 years).

**Figure 3:**
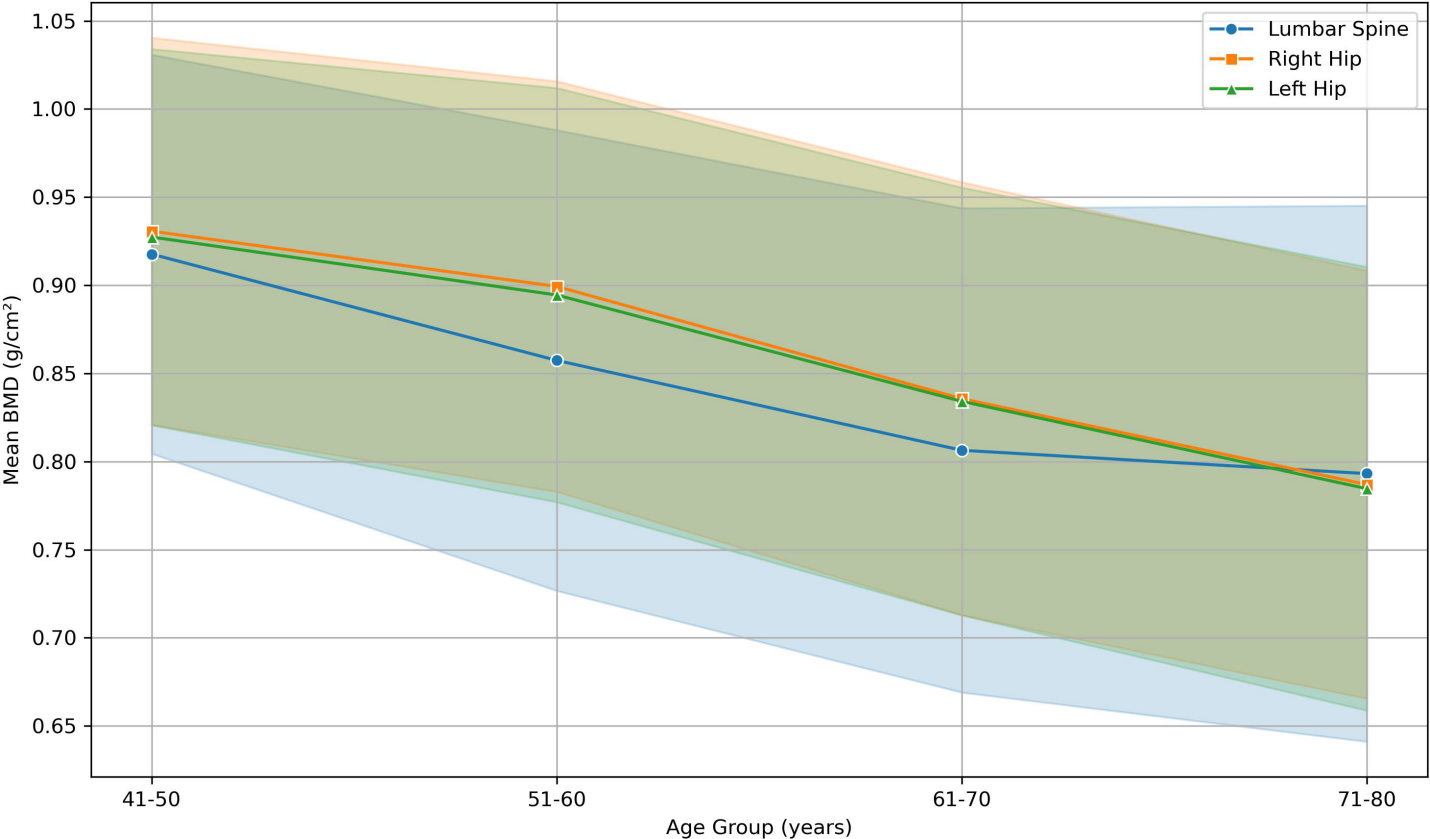
Age related trends in mean BMD at the lumbar spine and both hips among postmenopausal women.

Similar trends were observed in hip BMD. Pre-menopausal women had the highest means (right = 0.915–0.953 g/cm^2^; left = 0.913–0.951 g/cm^2^, ages 21–50), with a peak at 41–50 years. Early post-menopause (1–5 years) BMD in both hips showed a gradual loss of about 0.0043 g/cm^2^ to 0.0050 g/cm^2^ per year, continuing gradually through later stages. The lowest BMD occurred after >30 years since menopause (right = 0.746 g/cm^2^; left = 0.753 g/cm^2^ at ages 71–80). Welch’s ANOVA confirmed significant site-wise differences (right: F = 202.73, p < 0.001; left: F = 187.15, p < 0.001), with large *post-hoc* effects (Hedges’ g ≈ 1.6 right hip; 1.5 left hip). **Figure 3** presents the age -related trends in mean BMD at the lumbar spine and hip among postmenopausal women, illustrating a steeper decline in mean lumbar spine BMD in earlier post-menopausal age groups, and a more gradual but progressive reduction of mean hip BMD at later age groups.

### 3.3 Comparison with NHANES reference data

Comparisons with NHANES reference data (**Table 3**) showed that Sri Lankan men had significantly lower lumbar spine BMD across all age groups, with deficits ranging from −4.31% (31–40 years) to −10.20% (71–80 years) (all p < 0.05). Hip BMD was also lower, but the difference narrowed with age. Sri Lankan women similarly exhibited lower lumbar spine BMD than NHANES White females, particularly in older groups, with percentage differences from −8.45% (21–30 years) to −15.33% (61–70 years) (all p < 0.05). Hip BMD showed parallel trends, with significant reductions in both younger (21–40 years) and older (61–80 years) women. In contrast, among those aged 41–60 years (peri- and early post-menopause), mean hip BMD in the indexed study was marginally higher than the corresponding NHANES reference values; however, these differences were not statistically significant (p > 0.05).

**Table 3:** Comparison of age- and sex-specific lumbar spine, right hip, and left hip BMD values between the present study and National Health and Nutritional Examination Survey (NHANES) reference data.

Sex	Present study				NHANES (reference values)	Difference (%)		p - value	
Male	Age category	Sample Size	Mean (SD) g/cm^2^		Mean (SD) g/cm^2^	Lumbar spine		Lumbar spine	
	21-30	58	0.981 (0.128)		1.057 (0.110)	-0.076 (-7.19)		< 0.05	
	31-40	215	0.997 (0.130)		1.042 (0.117)	-0.045 (-4.31)		< 0.05	
	41-50	414	0.987 (0.120)		1.051 (0.129)	-0.064 (-6.08)		< 0.05	
	51-60	1786	0.965 (0.124)		1.053 (0.143)	-0.088 (-8.35)		< 0.05	
	61-70	1060	0.971 (0.137)		1.070 (0.142)	-0.099 (-9.25)		< 0.05	
	71-80	472	0.959 (0.152)		1.068 (0.177)	-0.109 (-10.20)		< 0.05	
Female	21-30	60	0.974 (0.130)		1.064 (0.106)	-0.090 (-8.45)		< 0.05	
	31-40	254	0.980 (0.119)		1.065 (0.110)	-0.085 (-7.98)		< 0.05	
	41-50	702	0.970 (0.128)		1.056 (0.134)	-0.086 (-8.14)		< 0.05	
	51-60	2485	0.870 (0.137)		0.993 (0.141)	-0.123 (-12.38)		< 0.05	
	61-70	2348	0.806 (0.137)		0.952 (0.142)	-0.146 (-15.33)		< 0.05	
	71-80	1092	0.793 (0.152)		0.902 (0.167)	-0.109 (-12.08)		< 0.05	
	Age category	Sample size	Mean (SD) g/cm^2^		Mean (SD) g/cm^2^	Difference (%)		p - value	
			Right hip	Left Hip		Right hip	Left hip	Right hip	Left hip
Male	21-30	58	0.976 (0.129)	0.966 (0.129)	1.067 (0.120)	-0.091 (-8.52)	-0.101 (-9.46)	< 0.05	< 0.05
	31-40	215	0.976 (0.128)	0.978 (0.129)	1.029 (0.134)	-0.053 (-5.15)	-0.051 (-4.95)	< 0.05	< 0.05
	41-50	414	0.978 (0.110)	0.980 (0.114)	1.040 (0.130)	-0.062 (-5.96)	-0.060 (-5.76)	< 0.05	< 0.05
	51-60	1786	0.984 (0.112)	0.981 (0.113)	1.015 (0.142)	-0.031 (-3.05)	-0.034 (-3.34)	< 0.05	< 0.05
	61-70	1060	0.968 (0.119)	0.964 (0.120)	0.997 (0.137)	-0.029 (-2.90)	-0.033 (-3.30)	< 0.05	< 0.05
	71-80	472	0.942 (0.130)	0.935 (0.127)	0.961 (0.143)	-0.019 (-1.97)	-0.026 (-2.70)	< 0.05	< 0.05
Female	21-30	60	0.915 (0.128)	0.913 (0.128)	0.971 (0.114)	-0.056 (-5.76)	-0.058 (-5.97)	< 0.05	< 0.05
	31-40	254	0.919 (0.118)	0.917 (0.116)	0.955 (0.125)	-0.036 (-3.76)	-0.038 (-3.97)	< 0.05	< 0.05
	41-50	702	0.948 (0.113)	0.945 (0.113)	0.944 (0.131)	+0.004 (0.42)	+0.001 (0.10)	> 0.05	> 0.05
	51-60	2485	0.904 (0.119)	0.900 (0.120)	0.893 (0.133)	+0.011 (1.23)	+0.007 (0.783)	> 0.05	> 0.05
	61-70	2348	0.835 (0.122)	0.834 (0.121)	0.852 (0.120)	-0.017 (-1.99)	-0.018 (-2.11)	< 0.05	< 0.05
	71-80	1092	0.786 (0.121)	0.784 (0.125)	0.802 (0.139)	-0.016 (-1.99)	-0.018 (-2.24)	< 0.05	< 0.05

## 4 Discussion

This large-scale cross-sectional study represents a broad analysis on age- and sex-related changes in BMD among Sri Lankan adults. Men consistently had higher mean lumbar spine and hip BMD compared with women throughout all age groups, similar to NHANES 1999–2004 (31) and 2005–2008 (30), which reported higher BMD in men across multiple skeletal sites. These findings confirm consistent trends across populations of inherently sex-related differences in skeletal mass, likely reflecting variations in bone size and hormonal influences.

In the current study, no significant differences were detected in lumbar spine BMD within females aged 21–50 years of age (p > 0.85; Hedges’ g < 0.1), reflecting stability in early adulthood. The peak lumbar BMD occurred in the 31–40-year age group (0.980 g/cm^2^) and declined significantly thereafter, with substantial losses beyond age 50 (g > 1.2 between ages 31–40 and 71–80). These results agree with previous studies performed in other populations (21, 24, 32, 33). In Indian populations, Makker et al. (24) and Aggarwal et al. (21) similarly recorded peak lumbar BMD in the 31–40 years of age group, though the BMD remained relatively stable until midlife, followed by a steady decline after menopause. While there was a slight decline in lumbar spine BMD between the 31–40 and 41–50 age groups in this study (mean difference: 0.0103 g/cm^2^), this change was not statistically significant (p = 0.856; Hedges’ g = 0.08); this may reflect limitations in the ability of cross-sectional studies to detect subtle premenopausal bone loss, which is more readily captured in other longitudinal studies (34, 35). However, the BMD fall from 51 to 80 years was statistically significant, thus indicating accelerated postmenopausal bone loss.

Peak lumbar spine BMD in men occurred at 31–40 years (0.997 g/cm^2^), similar to Indian data indicating peak values at similar ages (Makker et al. (24); Aggarwal et al. (21)). Despite variation in mean BMD across decades, the overall effect size for Welch’s ANOVA was small (F = 4.73, p < 0.001, η^2^ = 0.006), suggesting relative stability in male lumbar BMD with increasing age. Furthermore, the differences in BMD across age decades at the lumbar spine and hips were more pronounced in women than in men, as the pairwise comparisons demonstrated greater age-group differences in women which is consistent with other longitudinal studies reporting that women experience greater age-related BMD differences compared to men (31, 36, 37). An insignificant increase in male lumbar BMD across the 51–60 to 61–70-year interval (+0.006 g/cm^2^; p = 0.853; 95% CI = –0.124 to +0.033) would more likely be due to sampling variability than a real physiological trend. However, similar patterns of apparent maintenance or slight increases in BMD have been observed in Taiwanese (38), Singaporean (20), and Austrian (25) cohorts, possibly related to degenerative spinal changes including the formation of osteophytes, joint space narrowing and osteosclerosis in aging men.

In this study, right and left proximal femur BMD showed strong agreement, consistent with cadaveric evidence which found no significant side-to-side differences in geometry, density, or structural rigidity (39). Still, small individual asymmetries in structural stiffness can occur, highlighting the value of assessing both hips in clinical and research settings. Afzelius et al. (40) found slightly lower total hip BMD in the dominant side (p = 0.035), though no difference was seen at the femoral neck (p = 0.754). These findings support measuring BMD in both hips for precise screening and diagnosis, especially in postmenopausal women, to ensure proper preventive and therapeutic interventions.

Among females, hip BMD peaked between 41–50 years, showing significant increases from 31–40 years (right: +0.029 g/cm^2^, p = 0.0096; left: +0.028 g/cm^2^, p = 0.0107). Welch’s ANOVA also confirmed significant differences across age groups for both hips (right: *F* = 257.14, p < 1.18 × 10^-138^, η^2^ = 0.154; left: *F* = 243.44, p < 1.55 × 10^-133^, η^2^ = 0.149). This midlife plateau likely reflects a brief phase of skeletal consolidation before menopausal bone loss, possibly influenced by higher BMI and mechanical loading. After 50 years, BMD declined significantly (right: –0.043 g/cm^2^; left: –0.045 g/cm^2^ BMD loss between 41–50 and 51–60 years). This pattern is consistent with Korean National Health and Nutrition Examination Survey (41), which demonstrated that Korean women attained maximal BMD in the femoral neck, lumbar spine, and total hip during their 20s, 30s, and 40s, respectively, followed by a rapid decline after 50 years. In addition to that, another Korean population-based study by Cui et al. (42) also demonstrated that peak BMD in women occurred between 40–49 years for the femoral neck and trochanter. On the other hand, the study from the Indian population (24) reported an earlier total hip peak BMD in the 31–40 years age group. It should also be noted that most of the previous studies have analyzed hip subregions such as the femoral neck, trochanter, and Ward’s triangle individually, with the trochanter typically reaching peak BMD later, often in the 40s (42–44). As the present study focused on total hip BMD, future research should include subregional analyses to better characterize site-specific bone trends.

In men, both right and left hip BMD remained stable between 21–50 years, with no significant differences across early to mid-adulthood (all p ≥ 0.97; Hedges’ *g* ≤ 0.12). Mean differences between 21–30 and 31–40 years were minimal (right: +0.0006 g/cm^2^, p = 1.000; left: –0.0120 g/cm^2^, p = 0.989). Although Welch’s ANOVA indicated overall variation (right: *F* = 9.13, p < 2.8 × 10^-8^; left: *F* = 11.61, p < 1.4 × 10^-10^), *post-hoc* tests showed, these early adulthood increases were not statistically robust. Peak hip BMD appeared at 51–60 years (right: 0.9848 g/cm^2^; left: 0.9813 g/cm^2^), followed by notable declines after 60 years (right: –0.0424 g/cm^2^; left: –0.0459 g/cm^2^; both p < 3.6 × 10^-9^; Hedges’ *g* > 0.36), with the lowest BMD observed at 71–80 years. This suggests a delayed BMD peak in Sri Lankan men, with bone loss becoming evident after 60. Most previous studies have shown an earlier peak in male hip BMD at 20–30 years (24, 45) or 31–40 years (21). The later peak observed here may reflect lifestyle, BMI, or physical activity factors that help preserve bone mass into later decades. Previous research also supports a strong positive association between BMI and BMD, linking greater body mass with higher skeletal loading and bone strength (46, 47).

Among women, higher BMI consistently corresponded with greater BMD at all skeletal sites, with noticeable age-related decline only after 50 years. Up to midlife, lumbar and hip BMD remained comparable across BMI categories, whereas post-50 declines were significant. In men, age-related differences across BMI groups were minimal, indicating generally stable BMD patterns. Similar findings have been reported in earlier studies, which found a positive association between BMI and BMD in a nationally representative sample of older adults, with no variation by age, sex, or race (48). Consistent with these findings, Looker et al. (49) noted a significant interaction between race and overweight/obesity status in predicting osteoporosis prevalence among postmenopausal women, highlighting racial disparities in bone health outcomes. However, they did not find a significant age interaction, suggesting that BMI–BMD associations may vary by ethnicity and demographic context rather than age alone.

A significant inverse association was observed between BMD and years since menopause, confirmed by Welch’s ANOVA (F = 223.17, p < 8.82 × 10^-190^, η^2^ = 0.186), with menopausal duration accounting for almost 19% of the variance in lumbar BMD and 15 to 16% of hip BMD. Over the initial five postmenopausal years, lumbar spine BMD showed a decline of 0.0166 g/cm^2^ per year, slowing down thereafter at approximately 0.0094 g/cm^2^ per year. Hip BMD declined more gradually, around 0.0043 to 0.005 g/cm^2^ per year during early post menopause, but its decline accelerated later to 0.006 to 0.007 g/cm^2^ per year. The findings indicate well-described physiological patterns wherein trabecular-rich regions, like the spine, show early bone loss, while cortical-rich sites, such as the hip, are characterized by a more gradual but progressive decrease of BMD in later life (50–53).

Contralateral hip comparisons found statistically significant differences only in the youngest age group (21–30 years, p < 0.05), possibly due to early skeletal adaptation, habitual loading, or minor measurement variability. In the elderly, no significant side-to-side difference existed (p > 0.05), which supports earlier research showing strong anatomical symmetry in hip morphology and fracture patterns, especially in older adults (54, 55). From a practical perspective, this symmetry means that measuring just one hip could be sufficient in many cases. Such an approach could make BMD assessments more affordable and accessible, allowing more patients to undergo screening without compromising accuracy, particularly in settings with limited resources.

When compared with the NHANES white reference data, Sri Lankan participants exhibited consistent BMD deficits across all ages and sites, except at the hips of peri- and postmenopausal women, which showed nonsignificant differences. The largest deficits were in lumbar spine BMD of postmenopausal women, reaching up to 15% lower. These findings emphasize the importance of population-specific reference ranges to reduce the misclassification of osteoporosis risk. Similar efforts to establish ethnic-specific BMD databases have been undertaken in Indian (24), Taiwanese (56), Chinese (57), Japanese (58), Spanish (32), Swedish (33), and Italian (59) populations. Notably, Indian women have also been found to have lower mean spinal and hip BMD compared to North American and European reference populations (60, 61). Additionally, the World Health Organization (WHO) and the ISCD, 2019 have recognized the limitations of applying Western thresholds universally, calling for ethnicity-specific reference databases. Our findings also reinforce this recommendation and stress the urgent need for Sri Lankan population specific BMD standards to optimize diagnostic accuracy and treatment decisions.

While robust, the retrospective design and lack of some key lifestyle or biochemical variables, such as diet, vitamin D status, and physical activity, limit causal interpretation. The study focused on the Western Province, which may limit its generalizability to rural populations with different environmental and socioeconomic conditions, despite being justified by its high population density, diverse ethnic representation, and concentration of privately available central DXA facilities. Data were drawn from private healthcare institutions, as most of the DXA scanners are available only in the private healthcare institutions, providing high-quality imaging but potentially introducing selection bias, as individuals undergoing routine or self-referred screening may not represent the general population. Limited availability of DXA in public hospitals further restricts national representativeness. Future studies should incorporate broader geographic coverage once the facilities are more uniformly available, to develop more representative BMD reference values for Sri Lankan adults. Furthermore, in national hospitals, DXA is typically reserved for patients with clinically suspected osteoporosis, resulting in a skewed dataset that does not reflect the general population. Due to these constraints, assembling a large, representative cohort for normative reference development remains challenging. Additionally, the exclusion of subregional hip analyses (e.g., femoral neck, trochanter) limits the depth of clinical insight. Future studies should incorporate subregional skeletal analyses to develop more representative BMD reference values for the Sri Lankan population. Despite these limitations, the large sample size and rigorous exclusion criteria provide a robust foundation for deriving preliminary normative BMD reference values for Sri Lankan adults.

Building on these findings, future research from this study will incorporate advanced machine learning techniques, including random forests and neural networks, to uncover complex, nonlinear relationships that may not be detectable using traditional statistical methods. Additionally, exploring how lifestyle, nutrition, and genetic factors influence bone health could provide deeper insights into population-specific determinants of BMD. Ultimately, these efforts could guide personalized prevention strategies, improve early diagnosis, and strengthen public health initiatives for bone health in Sri Lanka and similar settings.

## 5 Conclusion

This study underlines the significant variability in BMD among populations and creates a need for Sri Lankan population specific reference values for the proper diagnosis of osteoporosis, assessment of risks, and planning at the public health level. With a large cohort (n = 10,946) across all adult age groups, and with central skeletal measurements obtained via DXA, this work represents one of the most comprehensive datasets on Sri Lankan bone health, offering greater diagnostic precision than studies using peripheral sites. The findings give added weight to the need for local BMD reference thresholds, rather than Western standards, to avoid diagnostic misclassification. Preventive screening should target middle-aged adults, as early BMD decline begins before advanced age. The strong weight/BMI and BMD association further highlights the importance of optimal nutrition and weight management in maintaining bone health.

## Data Availability

The datasets presented in this article are not readily available because of privacy and institutional restrictions. Data access may be granted by the corresponding author upon reasonable request and with permission from the participating healthcare institutions. Requests to access these datasets should be directed to hd_2024_01@med.pdn.ac.lk.
