# Supplementary material for: Sex and age specific bone mineral density trends in Sri Lankan adults support the need for normative reference data
Source: Front Endocrinol (Lausanne). 2026 Mar 4;17:1748490. doi: 10.3389/fendo.2026.1748490 (PMC12995607; doi:10.3389/fendo.2026.1748490)
Supplement: Supplementary file 1 [file DataSheet1.pdf]

QUESTIONNAIRE PRIOR TO DEXA SCAN

Patient ID	

Full Name ____________________

Date of Birth ____________________ Gender ____________________

Current Height (cm) ____________________ Weight (kg) ____________________

Ethnicity ____________________

Referring Physician ____________________

This information is required for a DEXA scan in order to provide a complete evaluation of the results to your referring physician. Individual patient results are compared to and correlated with results from other patients with similar ages and ethnic groups. PLEASE DIRECT ANY QUESTIONS YOU MAY HAVE TO THE TECHNOLOGIST PERFORMING YOUR DEXA SCAN.

1 (If female) are you pregnant ?

2 Have you had any radiological procedure (X- ray or CT) using the following agents within the last 7-10 days?

I. Iodine

II. Barium

3 Do you have any internal objects that could interfere with the scan, such as:

I. Pacemaker leads

II. Radioactive seeds

III. Metal implants

IV. Surgical staples

V. Foreign bodies, e.g. shrapnel

VI. Radio- opaque catheters or tubes

4 Are you wearing any objects in your hips and lower back area such as an ostomy device, metal buttons, snaps or jewellery (umbilical ring)?

5 Have you had any calcium supplements during the last 48 hours?

6 Have you had previous hip or vertebral fractures? ☐ Yes ☐ No

7 Have you had any fractures during your adult life which did not result from significant trauma (i.e. vehicle accident)? ☐ Yes ☐ No

8 Did either of your parents ever have a hip fracture? ☐ Yes ☐ No

9 Do you smoke? ☐ Yes ☐ No

10 Have you ever taken Glucocorticoids? ☐ Yes ☐ No

11 Do you have rheumatoid arthritis? ☐ Yes ☐ No

12 Do you have secondary osteoporosis ☐ Yes ☐ No

13 Do you drink 3 or more alcoholic drinks per day? ☐ Yes ☐ No

14 Are you being treated for osteoporosis? ☐ Yes ☐ No

15 Have you ever taken any of the following medications?

- ☐ Actonel (i.e. risedronate)
- ☐ Boniva (i.e. ibandronate)
- ☐ Evista (i.e raloxifene)
- ☐ Forteo (i.e. parathyroid hormone)
- ☐ Fosamax (i.e. alendronate)
- ☐ HRT (i.e. estrogen/ hormone therapy)
- ☐ Miacalcin (i.e. calcitonin)
- ☐ Protelos (i.e. strontium ranelate)
- ☐ Reclast (i.e. zoledronate)
- ☐ Prolia (i.e. denosumab)
- ☐ Vitamin D
- ☐ Calcium
- ☐ Other - (please specify) ____________________

16 Do you have any of the following medical conditions?

- ☐ Anorexia or Bulimia
- ☐ Any seizure disorders
- ☐ Asthma or Emphysema
- ☐ Cancer
- ☐ End stage renal disease
- ☐ Inflammatory bowel disease
- ☐ Hyperparathyroidism
- ☐ Hysterectomy
- ☐ Other - (Please specify) ____________________

17 What was your maximum height (inches)?

18 Do you perform weight bearing exercise regularly? ☐ Yes ☐ No

19 Do you regularly consume dairy products? ☐ Yes ☐ No

20 Do you drink caffeinated beverages? ☐ Yes ☐ No

If female

21 At what age did your menstrual period start? ________________Years.

22 Are you

☐ Pre menopausal

☐ Post- menopausal
If you are post menopausal, at what age did you attain menopause? __________Years

23 How many full term pregnancies have you had? ☐

24 Have you ever missed your menstrual period for more than 6 months in a row (not including pregnancy or menopause)?

☐ Yes ☐ No

I attest that the answers I have provided on this form are correct to the best of my knowledge and give consent for the DEXA scan. I have read and understood the contents of this form and I have had the opportunity to ask questions regarding the information on this form

____

PATIENT'S SIGNATURE

____

Date
