# Supplementary material for: Sex and age specific bone mineral density trends in Sri Lankan adults support the need for normative reference data
Source: Front Endocrinol (Lausanne). 2026 Mar 4;17:1748490. doi: 10.3389/fendo.2026.1748490 (PMC12995607; doi:10.3389/fendo.2026.1748490)
Supplement: Supplementary file 2 [file DataSheet2.pdf]

Female Left hip BMD Centile Reference Table

AGE	P5	P10	P25	P50	P75	P90	P95
21	0.847598	0.893429	0.979215	1.053516	1.141447	1.211137	1.252148
22	0.843126	0.888815	0.974149	1.048578	1.136410	1.206422	1.247620
23	0.838654	0.884201	0.969083	1.043639	1.131374	1.201707	1.243091
24	0.834183	0.879587	0.964017	1.038700	1.126337	1.196991	1.238563
25	0.829711	0.874972	0.958951	1.033762	1.121301	1.192276	1.234035
26	0.825239	0.870358	0.953885	1.028823	1.116264	1.187560	1.229507
27	0.820767	0.865744	0.948819	1.023884	1.111228	1.182845	1.224978
28	0.816296	0.861130	0.943753	1.018946	1.106191	1.178130	1.220450
29	0.811824	0.856516	0.938687	1.014007	1.101155	1.173414	1.215922
30	0.807352	0.851902	0.933621	1.009068	1.096118	1.168699	1.211394
31	0.802880	0.847288	0.928555	1.004130	1.091082	1.163983	1.206865
32	0.798409	0.842674	0.923489	0.999191	1.086045	1.159268	1.202337
33	0.793937	0.838060	0.918423	0.994252	1.081009	1.154552	1.197809
34	0.789465	0.833446	0.913357	0.989314	1.075972	1.149837	1.193281
35	0.784993	0.828832	0.908291	0.984375	1.070936	1.145122	1.188753
36	0.780522	0.824218	0.903225	0.979436	1.065899	1.140406	1.184224
37	0.776050	0.819604	0.898159	0.974498	1.060863	1.135691	1.179696
38	0.771578	0.814990	0.893093	0.969559	1.055826	1.130975	1.175168
39	0.767106	0.810375	0.888027	0.964620	1.050790	1.126260	1.170640
40	0.762635	0.805761	0.882961	0.959682	1.045753	1.121544	1.166111
41	0.758163	0.801147	0.877895	0.954743	1.040717	1.116829	1.161583
42	0.753691	0.796533	0.872829	0.949804	1.035680	1.112114	1.157055
43	0.749219	0.791919	0.867763	0.944865	1.030644	1.107398	1.152527
44	0.744747	0.787305	0.862697	0.939927	1.025607	1.102683	1.147998
45	0.740276	0.782691	0.857631	0.934988	1.020571	1.097967	1.143470
46	0.735804	0.778077	0.852565	0.930049	1.015534	1.093252	1.138942
47	0.731332	0.773463	0.847499	0.925111	1.010498	1.088536	1.134414
48	0.726860	0.768849	0.842433	0.920172	1.005461	1.083821	1.129885
49	0.722389	0.764235	0.837367	0.915233	1.000425	1.079106	1.125357
50	0.717917	0.759621	0.832301	0.910295	0.995388	1.074390	1.120829
51	0.713445	0.755007	0.827235	0.905356	0.990352	1.069675	1.116301
52	0.708973	0.750393	0.822169	0.900417	0.985315	1.064959	1.111772
53	0.704502	0.745778	0.817103	0.895479	0.980279	1.060244	1.107244

AGE	P5	P10	P25	P50	P75	P90	P95
54	0.700030	0.741164	0.812037	0.890540	0.975242	1.055529	1.102716
55	0.695558	0.736550	0.806972	0.885601	0.970206	1.050813	1.098188
56	0.691086	0.731936	0.801906	0.880663	0.965169	1.046098	1.093659
57	0.686615	0.727322	0.796840	0.875724	0.960133	1.041382	1.089131
58	0.682143	0.722708	0.791774	0.870785	0.955096	1.036667	1.084603
59	0.677671	0.718094	0.786708	0.865847	0.950060	1.031951	1.080075
60	0.673199	0.713480	0.781642	0.860908	0.945023	1.027236	1.075546
61	0.668728	0.708866	0.776576	0.855969	0.939987	1.022521	1.071018
62	0.664256	0.704252	0.771510	0.851030	0.934950	1.017805	1.066490
63	0.659784	0.699638	0.766444	0.846092	0.929914	1.013090	1.061962
64	0.655312	0.695024	0.761378	0.841153	0.924877	1.008374	1.057433
65	0.650841	0.690410	0.756312	0.836214	0.919841	1.003659	1.052905
66	0.646369	0.685795	0.751246	0.831276	0.914804	0.998943	1.048377
67	0.641897	0.681181	0.746180	0.826337	0.909768	0.994228	1.043849
68	0.637425	0.676567	0.741114	0.821398	0.904731	0.989513	1.039320
69	0.632953	0.671953	0.736048	0.816460	0.899695	0.984797	1.034792
70	0.628482	0.667339	0.730982	0.811521	0.894658	0.980082	1.030264
71	0.624010	0.662725	0.725916	0.806582	0.889622	0.975366	1.025736
72	0.619538	0.658111	0.720850	0.801644	0.884585	0.970651	1.021207
73	0.615066	0.653497	0.715784	0.796705	0.879549	0.965935	1.016679
74	0.610595	0.648883	0.710718	0.791766	0.874512	0.961220	1.012151
75	0.606123	0.644269	0.705652	0.786828	0.869476	0.956505	1.007623
76	0.601651	0.639655	0.700586	0.781889	0.864439	0.951789	1.003094
77	0.597179	0.635041	0.695520	0.776950	0.859403	0.947074	0.998566
78	0.592708	0.630427	0.690454	0.772012	0.854366	0.942358	0.994038
79	0.588236	0.625813	0.685388	0.767073	0.849330	0.937643	0.989510
80	0.583764	0.621198	0.680322	0.762134	0.844294	0.932928	0.984981

Female Lumbar spine BMD Centile Reference Table

AGE	P5	P10	P25	P50	P75	P90	P95
21	0.869045	0.927118	1.007595	1.079719	1.163112	1.231311	1.265476
22	0.863147	0.920805	1.001161	1.073643	1.157348	1.226261	1.261062
23	0.857249	0.914493	0.994728	1.067568	1.151584	1.221211	1.256647
24	0.851351	0.908180	0.988294	1.061492	1.145820	1.216161	1.252232
25	0.845453	0.901867	0.981861	1.055416	1.140056	1.211111	1.247817
26	0.839554	0.895555	0.975427	1.049341	1.134292	1.206061	1.243402
27	0.833656	0.889242	0.968994	1.043265	1.128528	1.201011	1.238988
28	0.827758	0.882929	0.962560	1.037190	1.122764	1.195961	1.234573
29	0.821860	0.876616	0.956127	1.031114	1.117000	1.190911	1.230158
30	0.815962	0.870304	0.949693	1.025039	1.111236	1.185861	1.225743
31	0.810064	0.863991	0.943260	1.018963	1.105472	1.180811	1.221329
32	0.804166	0.857678	0.936826	1.012887	1.099708	1.175761	1.216914
33	0.798268	0.851366	0.930393	1.006812	1.093944	1.170711	1.212499
34	0.792370	0.845053	0.923959	1.000736	1.088180	1.165661	1.208084
35	0.786472	0.838740	0.917526	0.994661	1.082416	1.160611	1.203670
36	0.780573	0.832428	0.911092	0.988585	1.076652	1.155561	1.199255
37	0.774675	0.826115	0.904659	0.982509	1.070888	1.150511	1.194840
38	0.768777	0.819802	0.898225	0.976434	1.065124	1.145461	1.190425
39	0.762879	0.813489	0.891792	0.970358	1.059360	1.140411	1.186011
40	0.756981	0.807177	0.885358	0.964283	1.053596	1.135361	1.181596
41	0.751083	0.800864	0.878925	0.958207	1.047832	1.130311	1.177181
42	0.745185	0.794551	0.872491	0.952132	1.042068	1.125261	1.172766
43	0.739287	0.788239	0.866058	0.946056	1.036304	1.120211	1.168352
44	0.733389	0.781926	0.859624	0.939980	1.030540	1.115161	1.163937
45	0.727491	0.775613	0.853191	0.933905	1.024776	1.110111	1.159522
46	0.721593	0.769300	0.846757	0.927829	1.019012	1.105061	1.155107
47	0.715694	0.762988	0.840324	0.921754	1.013248	1.100011	1.150693
48	0.709796	0.756675	0.833890	0.915678	1.007484	1.094961	1.146278
49	0.703898	0.750362	0.827457	0.909602	1.001720	1.089911	1.141863
50	0.698000	0.744050	0.821023	0.903527	0.995956	1.084861	1.137448
51	0.692102	0.737737	0.814590	0.897451	0.990192	1.079811	1.133034
52	0.686204	0.731424	0.808156	0.891376	0.984428	1.074761	1.128619
53	0.680306	0.725112	0.801723	0.885300	0.978664	1.069711	1.124204

AGE	P5	P10	P25	P50	P75	P90	P95
54	0.674408	0.718799	0.795289	0.879225	0.972900	1.064661	1.119789
55	0.668510	0.712486	0.788856	0.873149	0.967136	1.059611	1.115375
56	0.662612	0.706173	0.782423	0.867073	0.961372	1.054561	1.110960
57	0.656713	0.699861	0.775989	0.860998	0.955608	1.049511	1.106545
58	0.650815	0.693548	0.769556	0.854922	0.949844	1.044461	1.102130
59	0.644917	0.687235	0.763122	0.848847	0.944080	1.039411	1.097715
60	0.639019	0.680923	0.756689	0.842771	0.938316	1.034361	1.093301
61	0.633121	0.674610	0.750255	0.836695	0.932552	1.029311	1.088886
62	0.627223	0.668297	0.743822	0.830620	0.926788	1.024261	1.084471
63	0.621325	0.661985	0.737388	0.824544	0.921024	1.019211	1.080056
64	0.615427	0.655672	0.730955	0.818469	0.915260	1.014161	1.075642
65	0.609529	0.649359	0.724521	0.812393	0.909496	1.009111	1.071227
66	0.603631	0.643046	0.718088	0.806318	0.903732	1.004061	1.066812
67	0.597733	0.636734	0.711654	0.800242	0.897968	0.999011	1.062397
68	0.591834	0.630421	0.705221	0.794166	0.892204	0.993961	1.057983
69	0.585936	0.624108	0.698787	0.788091	0.886440	0.988911	1.053568
70	0.580038	0.617796	0.692354	0.782015	0.880676	0.983861	1.049153
71	0.574140	0.611483	0.685920	0.775940	0.874913	0.978811	1.044738
72	0.568242	0.605170	0.679487	0.769864	0.869149	0.973761	1.040324
73	0.562344	0.598857	0.673053	0.763788	0.863385	0.968711	1.035909
74	0.556446	0.592545	0.666620	0.757713	0.857621	0.963661	1.031494
75	0.550548	0.586232	0.660186	0.751637	0.851857	0.958611	1.027079
76	0.544650	0.579919	0.653753	0.745562	0.846093	0.953561	1.022665
77	0.538752	0.573607	0.647319	0.739486	0.840329	0.948511	1.018250
78	0.532853	0.567294	0.640886	0.733411	0.834565	0.943461	1.013835
79	0.526955	0.560981	0.634452	0.727335	0.828801	0.938411	1.009420
80	0.521057	0.554669	0.628019	0.721259	0.823037	0.933361	1.005006

Female Right hip BMD Centile Reference Table

AGE	P5	P10	P25	P50	P75	P90	P95
21	0.846065	0.902903	0.981374	1.066617	1.150996	1.224460	1.258633
22	0.841696	0.898061	0.976374	1.061455	1.145748	1.219335	1.253858
23	0.837326	0.893219	0.971374	1.056294	1.140500	1.214209	1.249084
24	0.832956	0.888377	0.966375	1.051133	1.135253	1.209083	1.244310
25	0.828587	0.883535	0.961375	1.045972	1.130005	1.203958	1.239536
26	0.824217	0.878693	0.956375	1.040811	1.124758	1.198832	1.234762
27	0.819848	0.873850	0.951375	1.035649	1.119510	1.193706	1.229988
28	0.815478	0.869008	0.946376	1.030488	1.114263	1.188581	1.225213
29	0.811108	0.864166	0.941376	1.025327	1.109015	1.183455	1.220439
30	0.806739	0.859324	0.936376	1.020166	1.103768	1.178329	1.215665
31	0.802369	0.854482	0.931377	1.015005	1.098520	1.173204	1.210891
32	0.798000	0.849640	0.926377	1.009843	1.093273	1.168078	1.206117
33	0.793630	0.844798	0.921377	1.004682	1.088025	1.162953	1.201342
34	0.789260	0.839956	0.916377	0.999521	1.082778	1.157827	1.196568
35	0.784891	0.835113	0.911378	0.994360	1.077530	1.152701	1.191794
36	0.780521	0.830271	0.906378	0.989198	1.072283	1.147576	1.187020
37	0.776152	0.825429	0.901378	0.984037	1.067035	1.142450	1.182246
38	0.771782	0.820587	0.896379	0.978876	1.061788	1.137324	1.177471
39	0.767413	0.815745	0.891379	0.973715	1.056540	1.132199	1.172697
40	0.763043	0.810903	0.886379	0.968554	1.051293	1.127073	1.167923
41	0.758673	0.806061	0.881379	0.963392	1.046045	1.121947	1.163149
42	0.754304	0.801218	0.876380	0.958231	1.040797	1.116822	1.158375
43	0.749934	0.796376	0.871380	0.953070	1.035550	1.111696	1.153601
44	0.745565	0.791534	0.866380	0.947909	1.030302	1.106571	1.148826
45	0.741195	0.786692	0.861381	0.942748	1.025055	1.101445	1.144052
46	0.736825	0.781850	0.856381	0.937586	1.019807	1.096319	1.139278
47	0.732456	0.777008	0.851381	0.932425	1.014560	1.091194	1.134504
48	0.728086	0.772166	0.846381	0.927264	1.009312	1.086068	1.129730
49	0.723717	0.767323	0.841382	0.922103	1.004065	1.080942	1.124955
50	0.719347	0.762481	0.836382	0.916942	0.998817	1.075817	1.120181
51	0.714977	0.757639	0.831382	0.911780	0.993570	1.070691	1.115407
52	0.710608	0.752797	0.826383	0.906619	0.988322	1.065566	1.110633
53	0.706238	0.747955	0.821383	0.901458	0.983075	1.060440	1.105859

AGE	P5	P10	P25	P50	P75	P90	P95
54	0.701869	0.743113	0.816383	0.896297	0.977827	1.055314	1.101084
55	0.697499	0.738271	0.811383	0.891135	0.972580	1.050189	1.096310
56	0.693129	0.733429	0.806384	0.885974	0.967332	1.045063	1.091536
57	0.688760	0.728586	0.801384	0.880813	0.962085	1.039937	1.086762
58	0.684390	0.723744	0.796384	0.875652	0.956837	1.034812	1.081988
59	0.680021	0.718902	0.791385	0.870491	0.951590	1.029686	1.077214
60	0.675651	0.714060	0.786385	0.865329	0.946342	1.024560	1.072439
61	0.671281	0.709218	0.781385	0.860168	0.941095	1.019435	1.067665
62	0.666912	0.704376	0.776385	0.855007	0.935847	1.014309	1.062891
63	0.662542	0.699534	0.771386	0.849846	0.930599	1.009184	1.058117
64	0.658173	0.694691	0.766386	0.844685	0.925352	1.004058	1.053343
65	0.653803	0.689849	0.761386	0.839523	0.920104	0.998932	1.048568
66	0.649434	0.685007	0.756387	0.834362	0.914857	0.993807	1.043794
67	0.645064	0.680165	0.751387	0.829201	0.909609	0.988681	1.039020
68	0.640694	0.675323	0.746387	0.824040	0.904362	0.983555	1.034246
69	0.636325	0.670481	0.741387	0.818879	0.899114	0.978430	1.029472
70	0.631955	0.665639	0.736388	0.813717	0.893867	0.973304	1.024698
71	0.627586	0.660797	0.731388	0.808556	0.888619	0.968178	1.019923
72	0.623216	0.655954	0.726388	0.803395	0.883372	0.963053	1.015149
73	0.618846	0.651112	0.721389	0.798234	0.878124	0.957927	1.010375
74	0.614477	0.646270	0.716389	0.793072	0.872877	0.952802	1.005601
75	0.610107	0.641428	0.711389	0.787911	0.867629	0.947676	1.000827
76	0.605738	0.636586	0.706390	0.782750	0.862382	0.942550	0.996052
77	0.601368	0.631744	0.701390	0.777589	0.857134	0.937425	0.991278
78	0.596998	0.626902	0.696390	0.772428	0.851887	0.932299	0.986504
79	0.592629	0.622059	0.691390	0.767266	0.846639	0.927173	0.981730
80	0.588259	0.617217	0.686391	0.762105	0.841392	0.922048	0.976956

Male Left hip BMD Centile Reference Table

AGE	P5	P10	P25	P50	P75	P90	P95
21	0.794503	0.857247	0.936768	1.020380	1.084721	1.161276	1.201411
22	0.793899	0.856231	0.935544	1.019022	1.083833	1.160280	1.200436
23	0.793296	0.855215	0.934321	1.017664	1.082946	1.159285	1.199461
24	0.792692	0.854199	0.933098	1.016306	1.082059	1.158290	1.198486
25	0.792089	0.853183	0.931874	1.014949	1.081172	1.157294	1.197511
26	0.791485	0.852167	0.930651	1.013591	1.080285	1.156299	1.196536
27	0.790882	0.851152	0.929428	1.012233	1.079397	1.155304	1.195561
28	0.790278	0.850136	0.928205	1.010875	1.078510	1.154308	1.194586
29	0.789675	0.849120	0.926981	1.009518	1.077623	1.153313	1.193610
30	0.789072	0.848104	0.925758	1.008160	1.076736	1.152317	1.192635
31	0.788468	0.847088	0.924535	1.006802	1.075848	1.151322	1.191660
32	0.787865	0.846073	0.923312	1.005444	1.074961	1.150327	1.190685
33	0.787261	0.845057	0.922088	1.004087	1.074074	1.149331	1.189710
34	0.786658	0.844041	0.920865	1.002729	1.073187	1.148336	1.188735
35	0.786054	0.843025	0.919642	1.001371	1.072300	1.147341	1.187760
36	0.785451	0.842009	0.918418	1.000013	1.071412	1.146345	1.186785
37	0.784847	0.840993	0.917195	0.998656	1.070525	1.145350	1.185810
38	0.784244	0.839978	0.915972	0.997298	1.069638	1.144355	1.184834
39	0.783641	0.838962	0.914749	0.995940	1.068751	1.143359	1.183859
40	0.783037	0.837946	0.913525	0.994582	1.067864	1.142364	1.182884
41	0.782434	0.836930	0.912302	0.993225	1.066976	1.141368	1.181909
42	0.781830	0.835914	0.911079	0.991867	1.066089	1.140373	1.180934
43	0.781227	0.834898	0.909855	0.990509	1.065202	1.139378	1.179959
44	0.780623	0.833883	0.908632	0.989151	1.064315	1.138382	1.178984
45	0.780020	0.832867	0.907409	0.987794	1.063428	1.137387	1.178009
46	0.779416	0.831851	0.906186	0.986436	1.062540	1.136392	1.177033
47	0.778813	0.830835	0.904962	0.985078	1.061653	1.135396	1.176058
48	0.778210	0.829819	0.903739	0.983720	1.060766	1.134401	1.175083
49	0.777606	0.828804	0.902516	0.982363	1.059879	1.133406	1.174108
50	0.777003	0.827788	0.901293	0.981005	1.058992	1.132410	1.173133
51	0.776399	0.826772	0.900069	0.979647	1.058104	1.131415	1.172158
52	0.775796	0.825756	0.898846	0.978289	1.057217	1.130420	1.171183
53	0.775192	0.824740	0.897623	0.976932	1.056330	1.129424	1.170208

AGE	P5	P10	P25	P50	P75	P90	P95
54	0.774589	0.823724	0.896399	0.975574	1.055443	1.128429	1.169233
55	0.773986	0.822709	0.895176	0.974216	1.054555	1.127433	1.168257
56	0.773382	0.821693	0.893953	0.972858	1.053668	1.126438	1.167282
57	0.772779	0.820677	0.892730	0.971501	1.052781	1.125443	1.166307
58	0.772175	0.819661	0.891506	0.970143	1.051894	1.124447	1.165332
59	0.771572	0.818645	0.890283	0.968785	1.051007	1.123452	1.164357
60	0.770968	0.817629	0.889060	0.967427	1.050119	1.122457	1.163382
61	0.770365	0.816614	0.887837	0.966070	1.049232	1.121461	1.162407
62	0.769761	0.815598	0.886613	0.964712	1.048345	1.120466	1.161432
63	0.769158	0.814582	0.885390	0.963354	1.047458	1.119471	1.160457
64	0.768555	0.813566	0.884167	0.961996	1.046571	1.118475	1.159481
65	0.767951	0.812550	0.882943	0.960639	1.045683	1.117480	1.158506
66	0.767348	0.811535	0.881720	0.959281	1.044796	1.116484	1.157531
67	0.766744	0.810519	0.880497	0.957923	1.043909	1.115489	1.156556
68	0.766141	0.809503	0.879274	0.956565	1.043022	1.114494	1.155581
69	0.765537	0.808487	0.878050	0.955208	1.042135	1.113498	1.154606
70	0.764934	0.807471	0.876827	0.953850	1.041247	1.112503	1.153631
71	0.764330	0.806455	0.875604	0.952492	1.040360	1.111508	1.152656
72	0.763727	0.805440	0.874381	0.951134	1.039473	1.110512	1.151681
73	0.763124	0.804424	0.873157	0.949777	1.038586	1.109517	1.150705
74	0.762520	0.803408	0.871934	0.948419	1.037699	1.108522	1.149730
75	0.761917	0.802392	0.870711	0.947061	1.036811	1.107526	1.148755
76	0.761313	0.801376	0.869487	0.945703	1.035924	1.106531	1.147780
77	0.760710	0.800361	0.868264	0.944346	1.035037	1.105535	1.146805
78	0.760106	0.799345	0.867041	0.942988	1.034150	1.104540	1.145830
79	0.759503	0.798329	0.865818	0.941630	1.033262	1.103545	1.144855
80	0.758899	0.797313	0.864594	0.940272	1.032375	1.102549	1.143880

Male Lumbar spine BMD Centile Reference Table

AGE	P5	P10	P25	P50	P75	P90	P95
21	0.828658	0.863295	0.922103	0.994058	1.071770	1.104295	1.158929
22	0.826758	0.861662	0.920977	0.993263	1.071450	1.105366	1.159923
23	0.824859	0.860029	0.919851	0.992469	1.071129	1.106437	1.160916
24	0.822960	0.858395	0.918724	0.991674	1.070808	1.107508	1.161909
25	0.821060	0.856762	0.917598	0.990879	1.070487	1.108579	1.162902
26	0.819161	0.855129	0.916472	0.990084	1.070166	1.109649	1.163896
27	0.817262	0.853496	0.915346	0.989290	1.069845	1.110720	1.164889
28	0.815362	0.851862	0.914219	0.988495	1.069524	1.111791	1.165882
29	0.813463	0.850229	0.913093	0.987700	1.069203	1.112862	1.166876
30	0.811564	0.848596	0.911967	0.986906	1.068882	1.113933	1.167869
31	0.809664	0.846963	0.910841	0.986111	1.068561	1.115004	1.168862
32	0.807765	0.845329	0.909714	0.985316	1.068240	1.116075	1.169856
33	0.805866	0.843696	0.908588	0.984522	1.067919	1.117146	1.170849
34	0.803966	0.842063	0.907462	0.983727	1.067598	1.118216	1.171842
35	0.802067	0.840430	0.906336	0.982932	1.067277	1.119287	1.172835
36	0.800168	0.838796	0.905209	0.982137	1.066957	1.120358	1.173829
37	0.798268	0.837163	0.904083	0.981343	1.066636	1.121429	1.174822
38	0.796369	0.835530	0.902957	0.980548	1.066315	1.122500	1.175815
39	0.794470	0.833897	0.901831	0.979753	1.065994	1.123571	1.176809
40	0.792570	0.832264	0.900704	0.978959	1.065673	1.124642	1.177802
41	0.790671	0.830630	0.899578	0.978164	1.065352	1.125713	1.178795
42	0.788772	0.828997	0.898452	0.977369	1.065031	1.126783	1.179788
43	0.786872	0.827364	0.897326	0.976575	1.064710	1.127854	1.180782
44	0.784973	0.825731	0.896200	0.975780	1.064389	1.128925	1.181775
45	0.783074	0.824097	0.895073	0.974985	1.064068	1.129996	1.182768
46	0.781174	0.822464	0.893947	0.974190	1.063747	1.131067	1.183762
47	0.779275	0.820831	0.892821	0.973396	1.063426	1.132138	1.184755
48	0.777376	0.819198	0.891695	0.972601	1.063105	1.133209	1.185748
49	0.775476	0.817564	0.890568	0.971806	1.062784	1.134280	1.186742
50	0.773577	0.815931	0.889442	0.971012	1.062464	1.135350	1.187735
51	0.771678	0.814298	0.888316	0.970217	1.062143	1.136421	1.188728
52	0.769778	0.812665	0.887190	0.969422	1.061822	1.137492	1.189721
53	0.767879	0.811031	0.886063	0.968628	1.061501	1.138563	1.190715

AGE	P5	P10	P25	P50	P75	P90	P95
54	0.765980	0.809398	0.884937	0.967833	1.061180	1.139634	1.191708
55	0.764080	0.807765	0.883811	0.967038	1.060859	1.140705	1.192701
56	0.762181	0.806132	0.882685	0.966243	1.060538	1.141776	1.193695
57	0.760282	0.804498	0.881558	0.965449	1.060217	1.142847	1.194688
58	0.758382	0.802865	0.880432	0.964654	1.059896	1.143918	1.195681
59	0.756483	0.801232	0.879306	0.963859	1.059575	1.144988	1.196674
60	0.754584	0.799599	0.878180	0.963065	1.059254	1.146059	1.197668
61	0.752684	0.797966	0.877053	0.962270	1.058933	1.147130	1.198661
62	0.750785	0.796332	0.875927	0.961475	1.058612	1.148201	1.199654
63	0.748886	0.794699	0.874801	0.960681	1.058291	1.149272	1.200648
64	0.746986	0.793066	0.873675	0.959886	1.057971	1.150343	1.201641
65	0.745087	0.791433	0.872548	0.959091	1.057650	1.151414	1.202634
66	0.743188	0.789799	0.871422	0.958296	1.057329	1.152485	1.203628
67	0.741288	0.788166	0.870296	0.957502	1.057008	1.153555	1.204621
68	0.739389	0.786533	0.869170	0.956707	1.056687	1.154626	1.205614
69	0.737490	0.784900	0.868043	0.955912	1.056366	1.155697	1.206607
70	0.735590	0.783266	0.866917	0.955118	1.056045	1.156768	1.207601
71	0.733691	0.781633	0.865791	0.954323	1.055724	1.157839	1.208594
72	0.731792	0.780000	0.864665	0.953528	1.055403	1.158910	1.209587
73	0.729893	0.778367	0.863538	0.952734	1.055082	1.159981	1.210581
74	0.727993	0.776733	0.862412	0.951939	1.054761	1.161052	1.211574
75	0.726094	0.775100	0.861286	0.951144	1.054440	1.162122	1.212567
76	0.724195	0.773467	0.860160	0.950349	1.054119	1.163193	1.213560
77	0.722295	0.771834	0.859033	0.949555	1.053798	1.164264	1.214554
78	0.720396	0.770200	0.857907	0.948760	1.053478	1.165335	1.215547
79	0.718497	0.768567	0.856781	0.947965	1.053157	1.166406	1.216540
80	0.716597	0.766934	0.855655	0.947171	1.052836	1.167477	1.217534

Male Right hip BMD Centile Reference Table

AGE	P5	P10	P25	P50	P75	P90	P95
21	0.808733	0.858513	0.936101	1.015103	1.088538	1.144652	1.169721
22	0.808009	0.857619	0.934945	1.014003	1.087674	1.144167	1.169619
23	0.807286	0.856725	0.933789	1.012904	1.086809	1.143681	1.169517
24	0.806563	0.855831	0.932632	1.011804	1.085945	1.143196	1.169414
25	0.805840	0.854936	0.931476	1.010704	1.085081	1.142711	1.169312
26	0.805117	0.854042	0.930319	1.009604	1.084217	1.142226	1.169210
27	0.804394	0.853148	0.929163	1.008504	1.083352	1.141740	1.169108
28	0.803671	0.852254	0.928007	1.007404	1.082488	1.141255	1.169006
29	0.802948	0.851359	0.926850	1.006304	1.081624	1.140770	1.168904
30	0.802225	0.850465	0.925694	1.005204	1.080760	1.140285	1.168802
31	0.801501	0.849571	0.924538	1.004104	1.079895	1.139799	1.168699
32	0.800778	0.848677	0.923381	1.003004	1.079031	1.139314	1.168597
33	0.800055	0.847783	0.922225	1.001904	1.078167	1.138829	1.168495
34	0.799332	0.846888	0.921068	1.000804	1.077303	1.138344	1.168393
35	0.798609	0.845994	0.919912	0.999704	1.076438	1.137858	1.168291
36	0.797886	0.845100	0.918756	0.998604	1.075574	1.137373	1.168189
37	0.797163	0.844206	0.917599	0.997504	1.074710	1.136888	1.168087
38	0.796440	0.843312	0.916443	0.996404	1.073846	1.136403	1.167984
39	0.795717	0.842417	0.915286	0.995304	1.072981	1.135917	1.167882
40	0.794994	0.841523	0.914130	0.994204	1.072117	1.135432	1.167780
41	0.794270	0.840629	0.912974	0.993104	1.071253	1.134947	1.167678
42	0.793547	0.839735	0.911817	0.992004	1.070388	1.134462	1.167576
43	0.792824	0.838840	0.910661	0.990904	1.069524	1.133976	1.167474
44	0.792101	0.837946	0.909504	0.989804	1.068660	1.133491	1.167372
45	0.791378	0.837052	0.908348	0.988704	1.067796	1.133006	1.167270
46	0.790655	0.836158	0.907192	0.987604	1.066931	1.132521	1.167167
47	0.789932	0.835264	0.906035	0.986504	1.066067	1.132035	1.167065
48	0.789209	0.834369	0.904879	0.985404	1.065203	1.131550	1.166963
49	0.788486	0.833475	0.903722	0.984304	1.064339	1.131065	1.166861
50	0.787763	0.832581	0.902566	0.983204	1.063474	1.130580	1.166759
51	0.787039	0.831687	0.901410	0.982104	1.062610	1.130094	1.166657
52	0.786316	0.830792	0.900253	0.981004	1.061746	1.129609	1.166555
53	0.785593	0.829898	0.899097	0.979904	1.060882	1.129124	1.166452

AGE	P5	P10	P25	P50	P75	P90	P95
54	0.784870	0.829004	0.897941	0.978804	1.060017	1.128639	1.166350
55	0.784147	0.828110	0.896784	0.977704	1.059153	1.128153	1.166248
56	0.783424	0.827216	0.895628	0.976604	1.058289	1.127668	1.166146
57	0.782701	0.826321	0.894471	0.975504	1.057425	1.127183	1.166044
58	0.781978	0.825427	0.893315	0.974404	1.056560	1.126698	1.165942
59	0.781255	0.824533	0.892159	0.973304	1.055696	1.126212	1.165840
60	0.780531	0.823639	0.891002	0.972204	1.054832	1.125727	1.165738
61	0.779808	0.822745	0.889846	0.971104	1.053968	1.125242	1.165635
62	0.779085	0.821850	0.888689	0.970004	1.053103	1.124757	1.165533
63	0.778362	0.820956	0.887533	0.968904	1.052239	1.124271	1.165431
64	0.777639	0.820062	0.886377	0.967804	1.051375	1.123786	1.165329
65	0.776916	0.819168	0.885220	0.966704	1.050511	1.123301	1.165227
66	0.776193	0.818273	0.884064	0.965604	1.049646	1.122816	1.165125
67	0.775470	0.817379	0.882907	0.964504	1.048782	1.122330	1.165023
68	0.774747	0.816485	0.881751	0.963404	1.047918	1.121845	1.164920
69	0.774024	0.815591	0.880595	0.962304	1.047054	1.121360	1.164818
70	0.773300	0.814697	0.879438	0.961204	1.046189	1.120875	1.164716
71	0.772577	0.813802	0.878282	0.960104	1.045325	1.120389	1.164614
72	0.771854	0.812908	0.877126	0.959004	1.044461	1.119904	1.164512
73	0.771131	0.812014	0.875969	0.957904	1.043597	1.119419	1.164410
74	0.770408	0.811120	0.874813	0.956804	1.042732	1.118934	1.164308
75	0.769685	0.810225	0.873656	0.955704	1.041868	1.118448	1.164206
76	0.768962	0.809331	0.872500	0.954604	1.041004	1.117963	1.164103
77	0.768239	0.808437	0.871344	0.953504	1.040139	1.117478	1.164001
78	0.767516	0.807543	0.870187	0.952404	1.039275	1.116993	1.163899
79	0.766792	0.806649	0.869031	0.951304	1.038411	1.116507	1.163797
80	0.766069	0.805754	0.867874	0.950204	1.037547	1.116022	1.163695
