# Supplementary material for: Sex and age specific bone mineral density trends in Sri Lankan adults support the need for normative reference data
Source: Front Endocrinol (Lausanne). 2026 Mar 4;17:1748490. doi: 10.3389/fendo.2026.1748490 (PMC12995607; doi:10.3389/fendo.2026.1748490)
Supplement: Supplementary file 3 [file Supplementaryfile1.docx]

Supplementary Material

# Supplementary Documents

## Document S1 - Structured questionnaire used to collect demographic and clinical information from participants.

## Document S2- Smoothed age- and sex-specific centile reference values (P5–P95) for lumbar spine, right hip, and left hip BMD. Centiles derived using Generalized Additive Models (GAM) presented in one-year increments for adults aged 21–80 years.

## Document S3- ERC and Institutional Authorization

# Supplementary Table

## Table S1 - Agreement between right and left hip BMD measurements. Comparative statistics assessing the concordance of BMD at the right and left hip across all age groups and sex.

| Sex | Mean Difference (g/cm²) | Lower Limit of Agreement (g/cm²) | Upper Limit of Agreement (g/cm²) | Interpretation |
| --- | --- | --- | --- | --- |
| Male | 0.0034 | –0.0798 | 0.0865 | Strong agreement; minimal bias |
| Female | 0.0029 | –0.0871 | 0.0930 | Strong agreement; minimal bias |

#
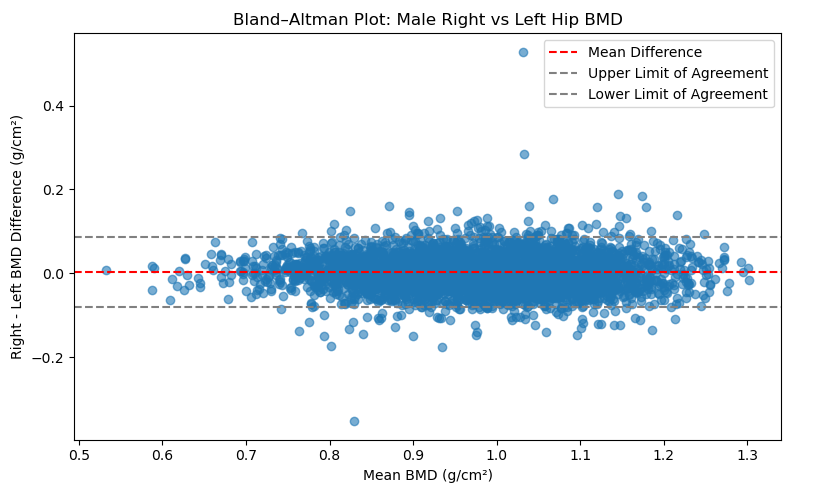
Figure S1. Bland–Altman plot comparing right versus left hip BMD in males.

# Figure S2. Bland–Altman plot comparing right versus left hip BMD in females.


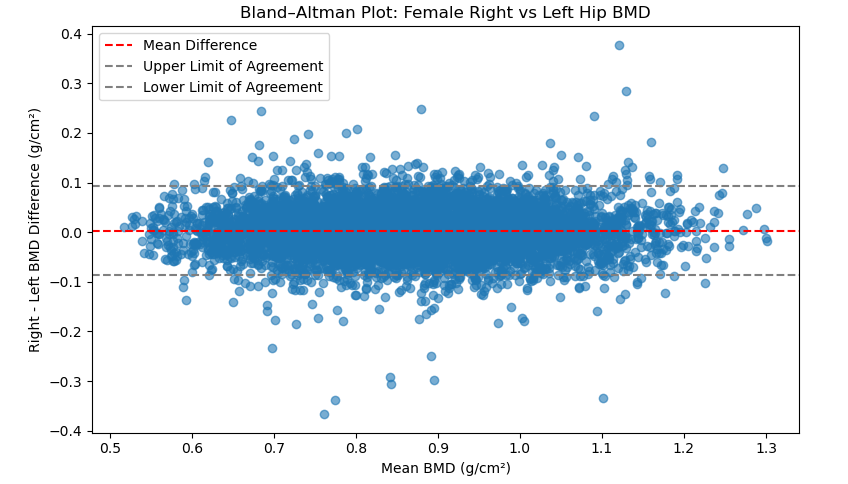


Legend - The plot shows the difference in BMD between the right and left hip against the mean BMD for each individual. **Red dashed line:** Mean difference between right and left hip BMD. **Black dashed lines**: Upper and lower limits of agreement. **Blue points:** Individual data points representing each subject’s BMD difference.

# Supplementary Figures

# Figure S3 (A-F). Age-related trends in BMD at the lumbar spine, left hip, and right hip in Sri Lankan males and females, modeled using Generalized Additive Models (GAMs).

1. Age-related trends in BMD at the lumbar spine (L1–L4) in Sri Lankan males.


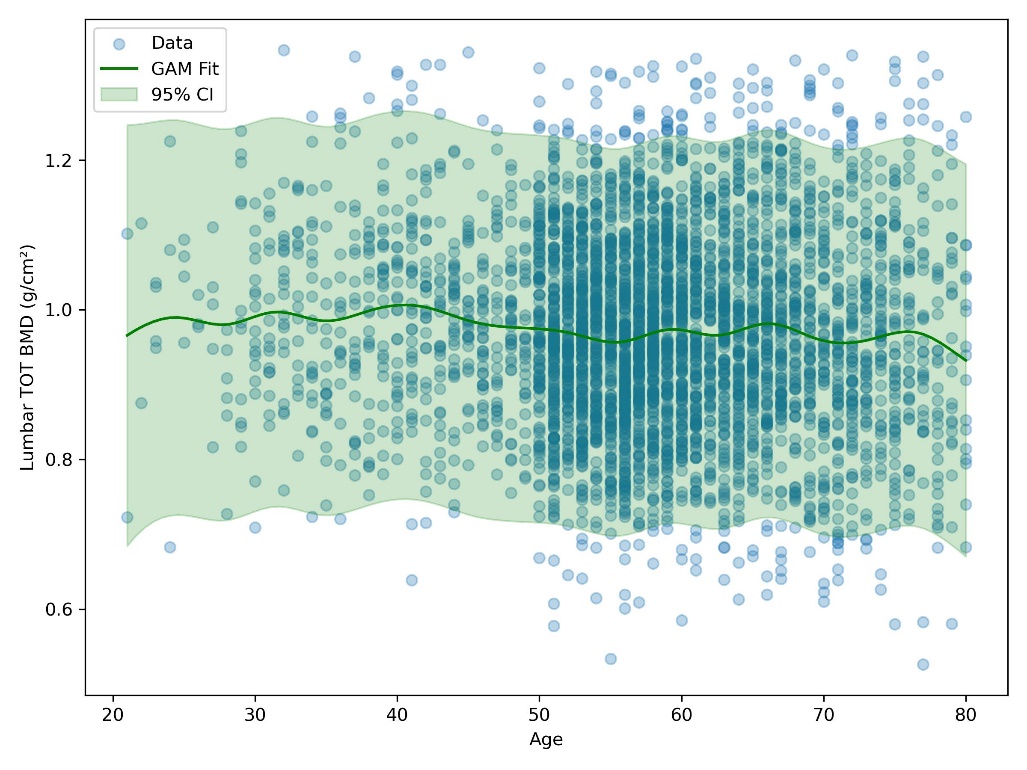


1. Age-related trends in BMD at the right hip in Sri Lankan males.


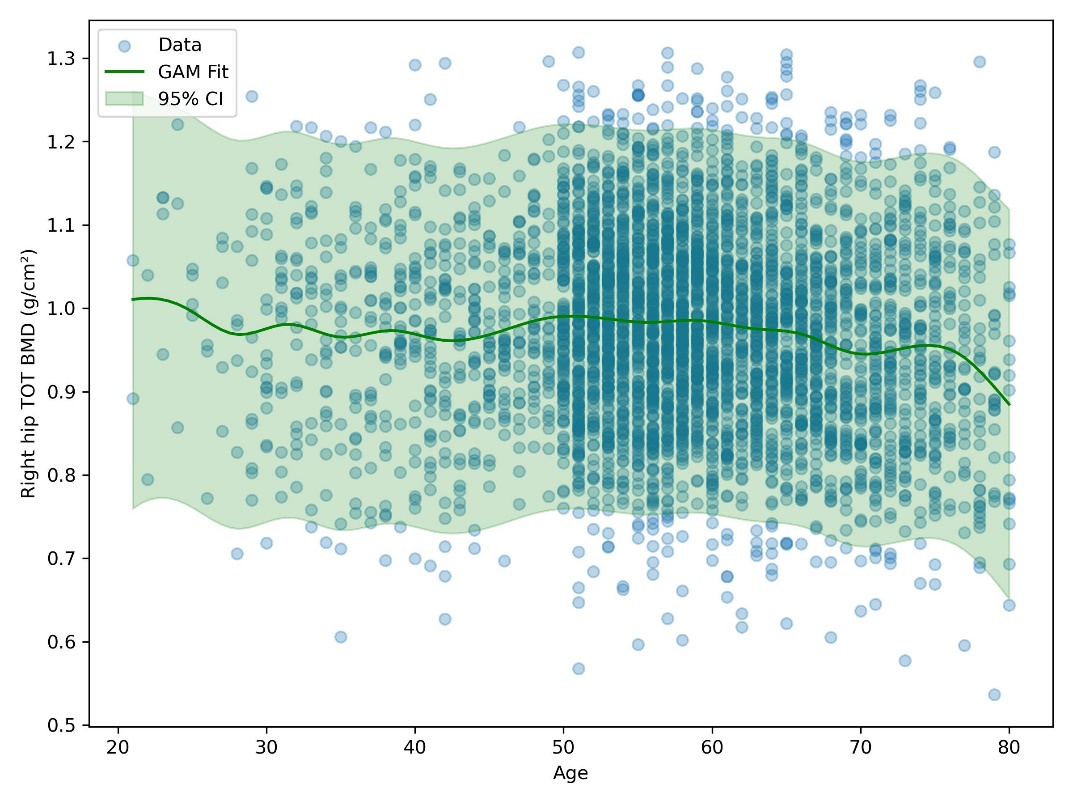


1. Age-related trends in BMD at the left hip in Sri Lankan males.


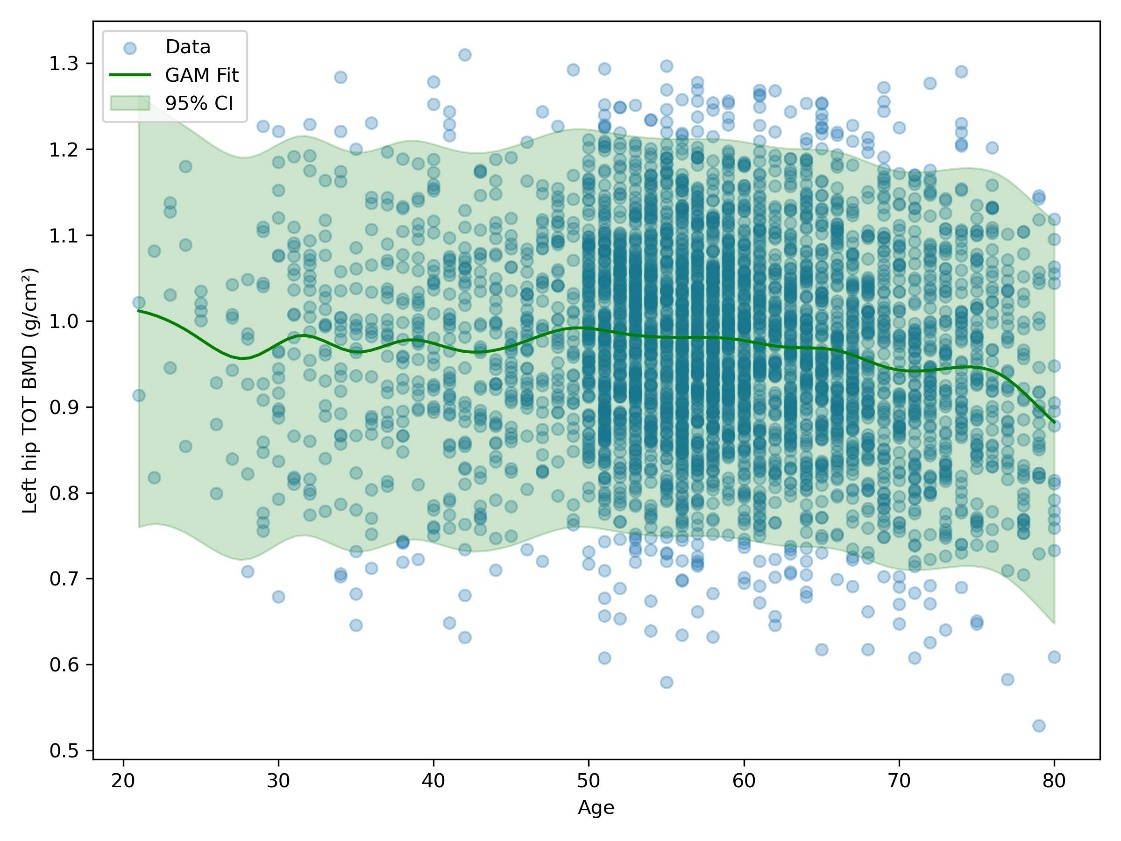


1. Age-related trends in BMD at the lumbar spine (L1–L4) in Sri Lankan females.


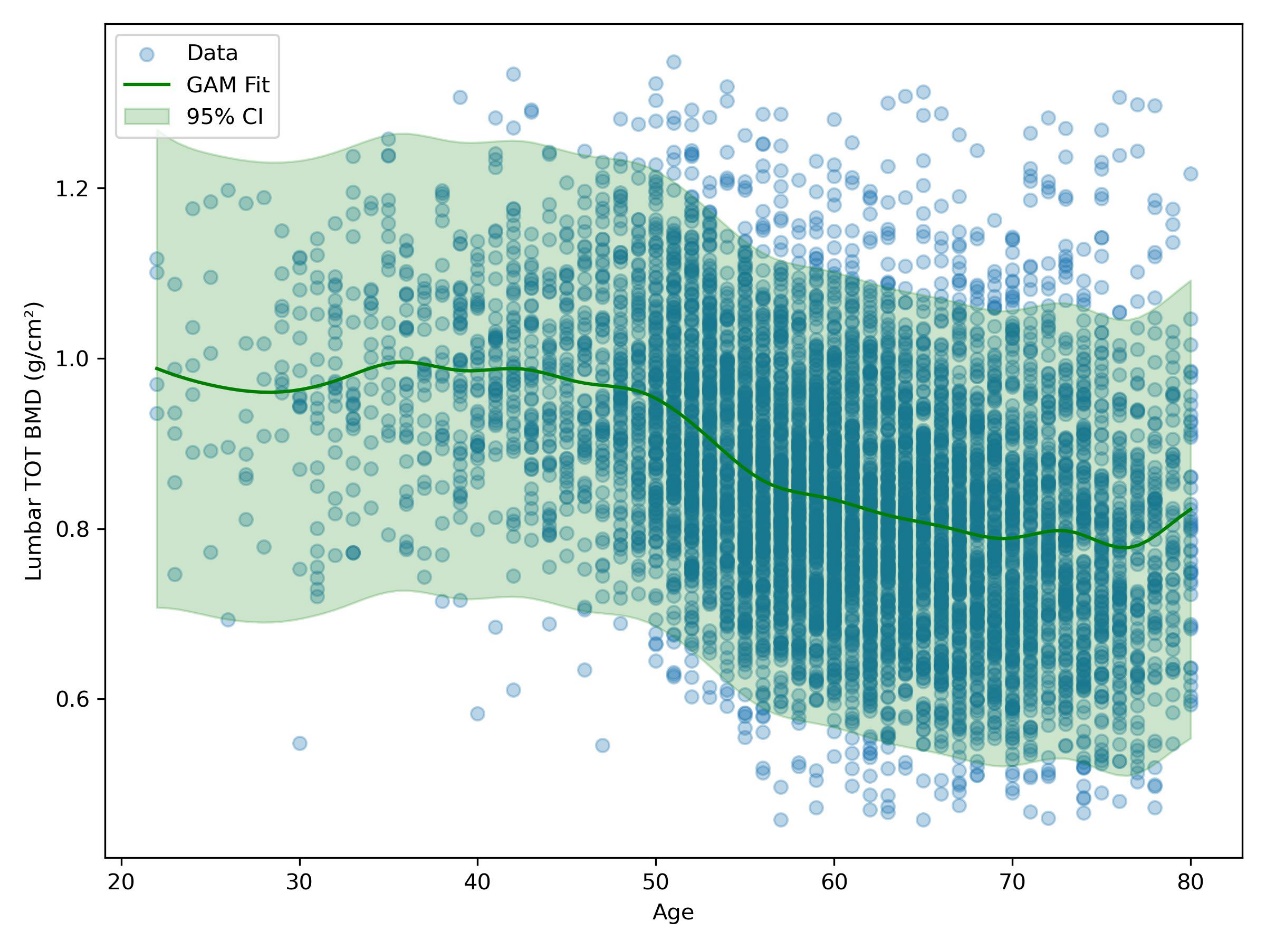


1. Age-related trends in BMD at the right hip in Sri Lankan females.


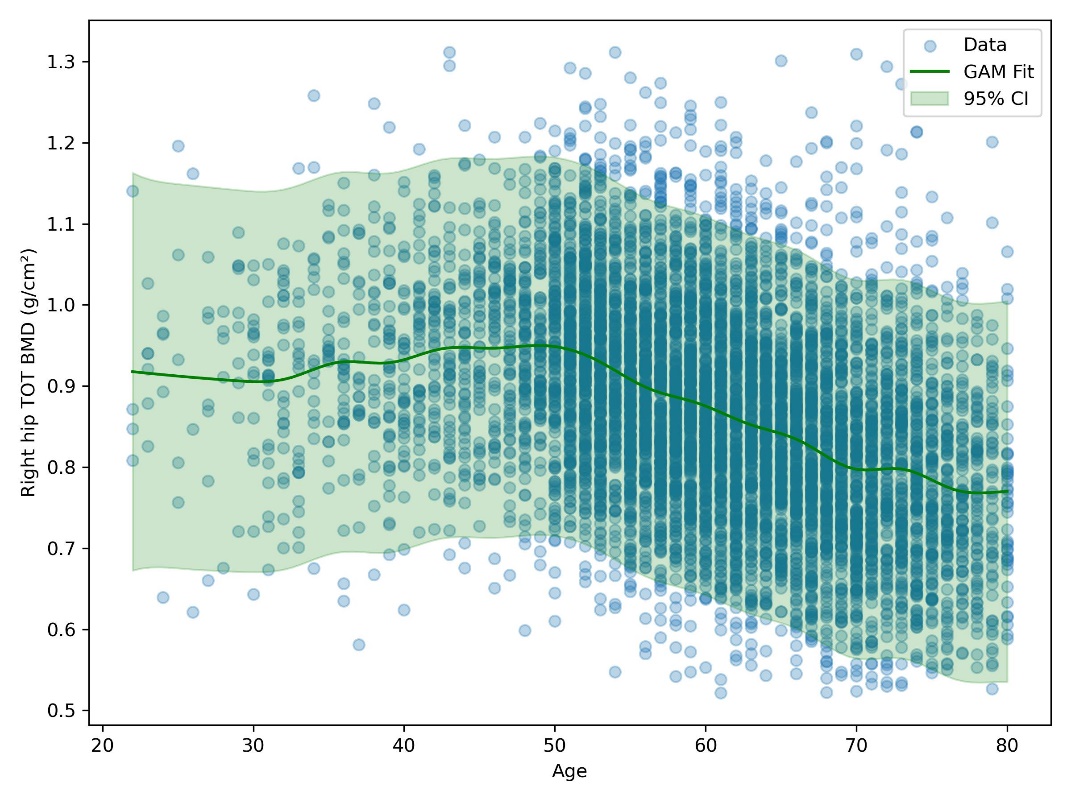


**(F)** Age-related trends in BMD at the left hip in Sri Lankan females.


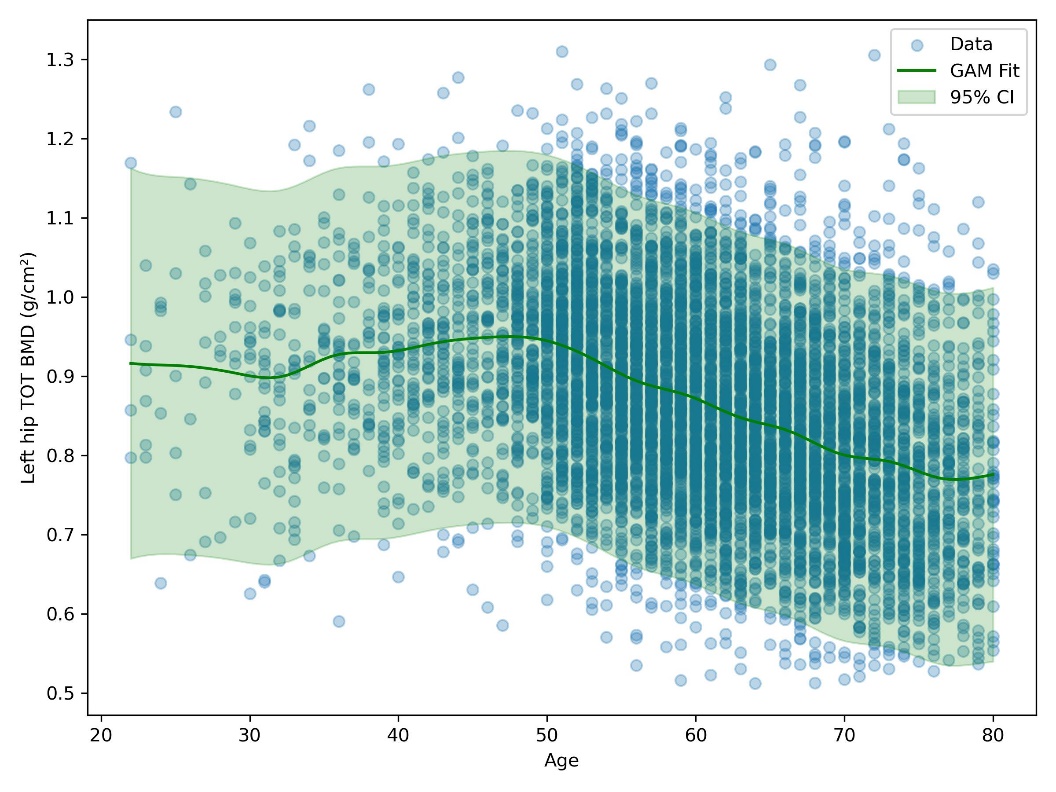


**Legend** The scatter plot shows individual lumbar total BMD measurements across ages 20–80 years. The solid green line represents the generalized additive model (GAM)–smoothed trend of BMD with age, while the shaded green area indicates the associated 95% confidence interval. The background distribution reflects variation of BMD values across the age span, demonstrating the percentile spread and overall pattern of bone density changes with ageing.
